# Cost Conversations About Anticoagulation Between Patients With Atrial Fibrillation and Their Clinicians: A Secondary Analysis of a Randomized Clinical Trial

**DOI:** 10.1001/jamanetworkopen.2021.16009

**Published:** 2021-07-13

**Authors:** Celia C. Kamath, Rachel Giblon, Marlene Kunneman, Alexander I. Lee, Megan E. Branda, Ian G. Hargraves, Angela L. Sivly, Fernanda Bellolio, Elizabeth A. Jackson, Bruce Burnett, Haeshik Gorr, Victor D. Torres Roldan, Gabriella Spencer-Bonilla, Nilay D. Shah, Peter A. Noseworthy, Victor M. Montori, Juan P. Brito

**Affiliations:** 1 Robert D. and Patricia E. Kern Center for the Science of HealthCare Delivery, Mayo Clinic, Rochester, Minnesota; 2 Knowledge and Evaluation Research Unit, Mayo Clinic, Rochester, Minnesota; 3 Division of Biomedical Statistics and Informatics, Department of Health Sciences Research, Mayo Clinic, Rochester, Minnesota; 4 Department of Biomedical Data Sciences, Leiden University Medical Center, Leiden, the Netherlands; 5 Colorado School of Public Health, Anschutz Medical Campus, University of Colorado, Denver, Aurora; 6 Department of Emergency Medicine, Mayo Clinic, Rochester, Minnesota; 7 Division of Cardiovascular Disease, University of Alabama at Birmingham, Birmingham; 8 Thrombosis Clinic and Anticoagulation Services, Park Nicollet Health Services, St Lois Park, Minnesota; 9 Division of General Internal Medicine, Hennepin Health, Minneapolis, Minnesota; 10 Stanford Internal Medicine Residency Program, California; 11 Heart Rhythm Services, Department of Cardiovascular Diseases, Mayo Clinic, Rochester, Minnesota; 12 Department of Endocrinology, Mayo Clinic, Rochester, Minnesota

## Abstract

**Importance:**

How patients with atrial fibrillation (AF) and their clinicians consider cost in forming care plans remains unknown.

**Objective:**

To identify factors that inform conversations regarding costs of anticoagulants for treatment of AF between patients and clinicians and outcomes associated with these conversations.

**Design, Setting, and Participants:**

This cohort study of recorded encounters and participant surveys at 5 US medical centers (including academic, community, and safety-net centers) from the SDM4AFib randomized trial compared standard AF care with and without use of a shared decision-making (SDM) tool. Included patients were considering anticoagulation treatment and were recruited by their clinicians between January 30, 2017, and June 27, 2019. Data were analyzed between August and November 2019.

**Main Outcomes and Measures:**

The incidence of and factors associated with cost conversations, and the association of cost conversations with patients’ consideration of treatment cost burden and their choice of anticoagulation.

**Results:**

A total of 830 encounters (out of 922 enrolled participants) were recorded. Patients’ mean (SD) age was 71.0 (10.4) years; 511 patients (61.6%) were men, 704 (86.0%) were White, 303 (40.9%) earned between $40 000 and $99 999 in annual income, and 657 (79.2%) were receiving anticoagulants. Clinicians’ mean (SD) age was 44.8 (13.2) years; 75 clinicians (53.2%) were men, and 111 (76%) practiced as physicians, with approximately half (69 [48.9%]) specializing in either internal medicine or cardiology. Cost conversations occurred in 639 encounters (77.0%) and were more likely in the SDM arm (378 [90%] vs 261 [64%]; OR, 9.69; 95% CI, 5.77-16.29). In multivariable analysis, cost conversations were more likely to occur with female clinicians (66 [47%]; OR, 2.85; 95% CI, 1.21-6.71); consultants vs in-training clinicians (113 [75%]; OR, 4.0; 95% CI, 1.4-11.1); clinicians practicing family medicine (24 [16%]; OR, 12.12; 95% CI, 2.75-53.38]), internal medicine (35 [23%]; OR, 3.82; 95% CI, 1.25-11.70), or other clinicians (21 [14%]; OR, 4.90; 95% CI, 1.32-18.16) when compared with cardiologists; and for patients with an annual household income between $40 000 and $99 999 (249 [82.2%]; OR, 1.86; 95% CI, 1.05-3.29) compared with income below $40 000 or above $99 999. More patients who had cost conversations reported cost as a factor in their decision (244 [89.1%] vs 327 [69.0%]; OR 3.66; 95% CI, 2.43-5.50), but cost conversations were not associated with the choice of anticoagulation agent.

**Conclusions and Relevance:**

Cost conversations were common, particularly for middle-income patients and with female and consultant-level primary care clinicians, as well as in encounters using an SDM tool; they were associated with patients’ consideration of treatment cost burden but not final treatment choice. With increasing costs of care passed on to patients, these findings can inform efforts to promote cost conversations in practice.

**Trial Registration:**

ClinicalTrials.gov Identifier: NCT02905032

## Key Points

**Question:**

What factors contribute to cost conversations about anticoagulation treatment between patients with atrial fibrillation and their clinicians, and what outcomes are associated with these conversations?

**Findings:**

In this cohort study of 830 audiovisual recordings of encounters and participant surveys from a randomized trial comparing atrial fibrillation care with and without a shared decision-making (SDM) tool, cost conversations were associated with the use of an SDM tool, with middle-income patients, and with consultations conducted by female primary care staff clinicians. Cost conversations were associated with patients’ decision-making processes but not final treatment choice.

**Meaning:**

These findings suggest that SDM tools may inform efforts to promote cost conversations in practice, an important consideration when increasing costs of care are being passed on to patients.

## Teaser

This cohort study, a secondary analysis of a randomized clinical trial, assesses factors contributing to cost conversations about care plans between clinicians and patients with atrial fibrillation.

## Introduction

Atrial fibrillation (AF) affects over 5 million people in the US.^1,2^ AF may reduce the quality and duration of life, particularly by increasing the risk of devastating thromboembolic strokes, and it accounts for over $25 billion a year in health care costs, mostly from preventable thromboembolic strokes.^3^ Use of oral anticoagulants, warfarin, and non-vitamin K antagonists or direct oral anticoagulants (DOACs) prevents strokes in patients with AF. Yet up to half of all patients with AF do not receive oral anticoagulants,^4^ and of those who do initiate treatment, 30% to 50% will discontinue therapy within the first 12 months.^5,6^

Treatment costs burden patients directly, whether through out-of-pocket costs such as deductibles, copayments, and coinsurance for those who are insured, or the total cost of medications for those that are not insured. With 1 in 3 patients having trouble paying their medical bills,^7^ delays, avoidance of care, and nonadherence expose patients to the risk of preventable strokes.^4,8^

Still, cost conversations occur infrequently in practice: only 1 in 3 clinicians report ever having such conversations with their patients.^9^ Cost conversations should be part of the work patients and clinicians do to co-create sensible and feasible treatment programs, work that is often called shared decision-making (SDM). Addressing the cost of treatment options at the point of prescription can result in feasible care, lessening the need for future discovery and remediation of cost-related adherence issues. These cost conversations during the clinical encounter may enable patients to make choices more closely aligned with their personal financial circumstances and preferences. Except for 2 studies, one in surgical patients^10^ and another in patients with several medical conditions,^11^ there is limited research on the ability of SDM tools to promote and support conversations about treatment costs.

Current guidelines suggest the use of DOACs over warfarin^12^ because DOACs are somewhat safer^13^ and do not need regular blood draws for International Normalized Ratio (INR) monitoring. However, DOACs are more expensive to patients relative to warfarin,^14,15^ partly accounting for the low initiation and adherence rates to DOACs.^16,17,18^ On the other hand, warfarin entails indirect costs such as time and travel for INR testing, costs of laboratory tests, etc.

In the SDM4AFib multicenter trial, we demonstrated that using an SDM conversation tool designed for use during the clinical encounter improved aspects of SDM quality and clinician satisfaction.^19^ This SDM tool included information on the direct annual cost estimates of each treatment option presented to patients. To the extent that the tool highlighted cost (among other patient important issues such as bleeding risk, etc), the SDM tool may have encouraged cost conversations. The present secondary analysis sought to assess the association of using this effective SDM tool with (1) the incidence of cost conversations between patients with AF and their clinicians and (2) patient, clinician, and encounter factors associated with their occurrence; we also sought to estimate the degree to which these cost conversations were associated with the choice of anticoagulation agent.

## Methods

### Study Design

This is a secondary analysis of audiovisual recordings of clinical encounters and patient surveys obtained during the conduct of the Shared Decision Making for Atrial Fibrillation (SDM4AFib) trial, an encounter-randomized multicentered trial comparing standard care with and without the use of an SDM encounter tool. The trial protocol and its primary outcomes have been published elsewhere (trial protocol available in Supplement 1).^19,20^ Institutional review boards at the coordinating center (ie, the Mayo Clinic) and at participating sites approved all trial procedures. Specifically, recordings took place when both patients and clinicians gave written informed consent. This study followed the Strengthening the Reporting of Observational Studies in Epidemiology (STROBE) reporting guideline for observational studies.

### Setting and Participants

The trial took place in 5 (academic, community, and safety-net) medical centers in the Midwest (3), Alabama (1), and Mississippi (1). The practice settings within these centers included emergency departments, outpatient safety-net facilities, primary care and cardiology clinics, and inpatient hospital services. Participants included patients and clinicians. Adult patients (ie, at least age 18 years) were eligible if they had a diagnosis of nonvalvular AF, were at high risk of a thromboembolic event (ie, had a CHA_2_DS_2_-VASc [congestive heart failure, hypertension, age 75 years or older, diabetes, stroke or transient ischemic attack, vascular disease, age 65 to 74 years, sex] score of 1 or greater in men and 2 or greater in women), and were able to read and understand the informed consent document. Patients were enrolled into 2 cohorts: a start cohort including patients new to anticoagulation and a review cohort of patients receiving ongoing anticoagulation. All clinicians at the participating medical centers who regularly had conversations with patients with AF about anticoagulation were eligible for participation. Clinicians completed a baseline survey at the time of study enrollment. Data were collected from both patients and clinicians immediately following the clinical encounter: the postencounter survey captured patient demographics, health literacy, subjective numeracy, and medication and treatment outcomes.

### Intervention

The Anticoagulation Choice SDM encounter tool^21^ first provides the patient’s tailored stroke risk at 1 or 5 years with and without anticoagulation using the CHA_2_DS_2_-VASc score. Then, the tool compares treatment options by patient-important issues, such as medication use, risk of bleeding (using the HAS-BLED [hypertension, abnormal kidney or liver function, stroke, bleeding, labile international normalized ratio, elderly age {over 65 years}, and drug of alcohol use] estimator),^22^ the need for periodic monitoring, reversibility, and estimated out-of-pocket costs. Data from a public web service were used to offer cost estimates for each treatment option.^23^ A systematic review was the source of estimates of the cost of monitoring the INR to adjust warfarin doses^24^ (eFigure in Supplement 2).

### Outcomes

The outcome for this secondary analysis was the occurrence of a cost conversation in the encounter. Examples of cost conversations included any statement or verbal exchange between clinician and patient or caregiver about cost or other financial considerations surrounding anticoagulation agents, including indirect costs, insurance-related costs and offers of financial assistance. This conversation could be brief (eg, mere mention of costs being important) or lengthy (eg, extensive discussion on patient possibly entering the Medicare Part D “donut hole” if the patient did not have supplemental drug coverage). Cost conversations could take place altogether during the consultation or take place intermittently throughout the encounter.

Two reviewers, after training and documentation of reliability, worked independently to ascertain whether or not a cost conversation occurred. Chance-adjusted inter-rater reliability (using Lin’s concordance correlation coefficient) was verified at the start, and again a third and two-thirds into the process of reviewing all recordings; this ranged from 0.84 to 0.96. We also used patients’ electronic medical records to note which anticoagulation agent (warfarin vs DOAC, other decision) was selected. In postencounter surveys, patients reported whether “cost did not matter,” “cost was one factor in my decision,” or “cost was the sole factor in my decision.”

### Patient and Clinician Factors

We were interested in exploring the extent to which patient age, sex, race/ethnicity (ie, White vs non-White race, Hispanic vs non-Hispanic ethnicity), marital status (married vs other), highest level of schooling (high school graduate or less vs more than high school graduate vs other), stroke risk (low vs high), medication cohort (start vs review), and total number of medications taken were associated with the incidence of cost conversations. Marital status was included as it was assumed to be associated with financial means. Race was included because it was assumed to affect the need and comfort level of cost conversations.

We also explored associations between the incidence of cost conversations and clinician characteristics, including age, sex, training status (staff vs in-training), patients seen per week, clinician type (Medical Doctor [MD] or Doctor of Ostheopathic Medicine [DO] vs other [eg, physician assistant, nurse practitioner, nurse]), medical specialty (cardiology [general or electrophysiology], family medicine, internal medicine, or other), years in practice (4 or less [median] vs more than 4 years), experience feelings of burned out (once a week or less vs a few times a week or daily). We captured this information from preencounter surveys completed by clinicians participating in the study.

### Statistical Analysis

Our primary analysis was conducted at the encounter level using multilevel mixed-effects models adjusting for study arm and patient characteristics, with random effects allowed for clinics and clinicians within medical centers^25^ and in accordance with the intention-to-treat principle, that is, analyzing encounters in the arm to which they were randomized. We also conducted a per-protocol analysis based on whether the SDM tool was actually used in the encounter. As we did not assume that patients and clinicians declined being recorded at random, we compared the participant characteristics of those with available recorded encounters with the characteristics of participants in all encounters. We tested univariate associations between cost conversations and patient, clinician, and encounter characteristics using χ^2^ and nonparametric 2-sample Kruskall-Wallis tests. Multivariable mixed-effects logistic regression included independent variables found to be significant in bivariate analysis and additional variables purported to influence conversations on costs (eg, income, education, marital status).^11,26,27^ We also tested univariate associations between the occurrence of cost conversation in the encounter and (1) the choice of anticoagulation agent, and (2) whether or not cost was a factor in medication decision-making. We used SAS software version 9.4 (SAS Institute) to conduct the analyses. *P* < .05 was considered statistically significant in 2-sided tests.

## Results

The Figure describes the flow of participants and data. We obtained audiovisual recordings in 830 of the 922 (90.0%) encounters in the SDM4AFib trial across the 5 medical centers. The mean (SD) age of patients included in this analysis was 71.0 (10.4) years, 511 participants (61.6%) were men, 704 (86.0%) were White, 303 (40.9%) reported income in the $40 000 to $99 999 range, and most patients (79%) were already taking an anticoagulation agent (Table 1). Patient characteristics were similar between the complete cohort and those with recordings.

**Figure.**
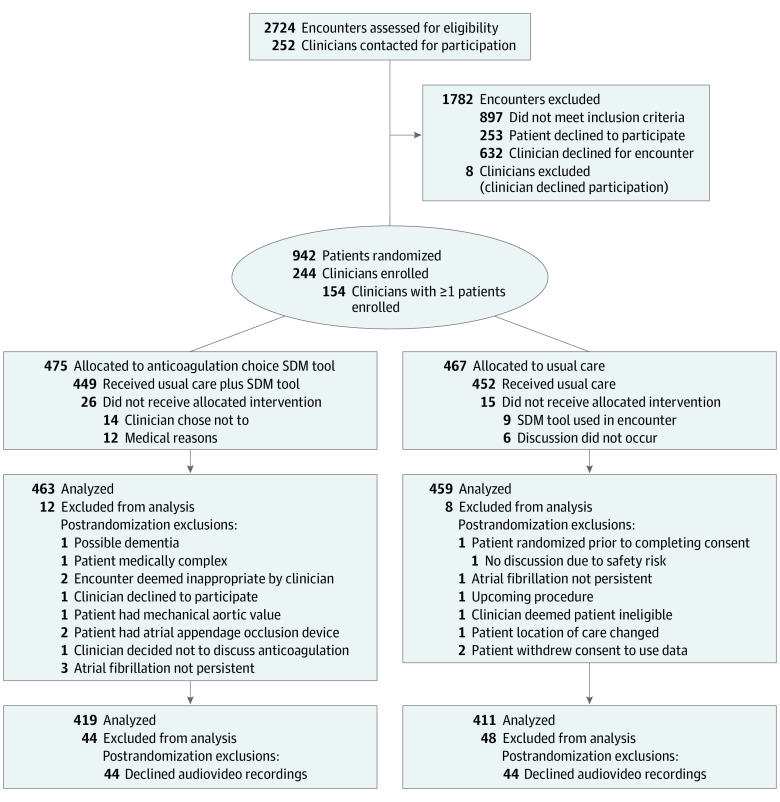
CONSORT Flow Diagram

**Table 1.** Patient Characteristics

Characteristics	Patients, No. (%)			
	Total (n = 922)	With video or audio encounters (n = 830)	Had cost conversation	
			No (n = 191)	Yes (n = 639)
Arm				
Standard care	459 (49.8)	411 (49.5)	150 (36.5)	261 (63.5)
Intervention arm (SDM tool)	463 (50.2)	419 (50.5)	41 (9.8)	378 (90.2)
Patient demographics				
Age, mean (SD), y	70.8 (10.4)	71.0 (10.4)	69.3 (10.4)	71.5 (10.4)
Gender				
Female	363 (39.4)	319 (38.4)	68 (21.3)	251 (78.7)
Male	559 (60.6)	511 (61.6)	123 (24.1)	388 (75.9)
White/Caucasian race^a^				
No	128 (14.1)	115 (14.0)	32 (27.8)	83 (72.2)
Yes	780 (85.9)	704 (86.0)	155 (22.0)	549 (78.0)
Ethnicity				
Not Hispanic or Latino	886 (98.7)	801 (98.8)	183 (22.8)	618 (77.2)
Hispanic or Latino	7 (0.8)	6 (0.7)	1 (16.7)	5 (83.3)
Stroke risk				
Low	139 (15.1)	116 (14.0)	36 (31.0)	80 (69.0)
High	783 (84.9)	714 (86.0)	155 (21.7)	559 (78.3)
Medication cohort				
Start	206 (22.3)	173 (20.8)	40 (23.1)	133 (76.9)
Review	716 (77.7)	657 (79.2)	151 (23.0)	506 (77.0)
Marital status				
Married	535 (59.3)	479 (58.8)	120 (25.1)	359 (74.9)
Other	367 (40.7)	335 (41.2)	65 (19.4)	270 (80.6)
Highest level of schooling				
High school graduate or less	212 (24.5)	191 (24.4)	45 (23.6)	146 (76.4)
4-year degree or some college	483 (55.8)	438 (55.9)	101 (23.1)	337 (76.9)
Graduate or professional school	170 (19.7)	155 (19.8)	30 (19.4)	125 (80.6)
Household income, $				
<40 000	289 (35.6)	265 (35.6)	62 (23.4)	203 (76.6)
40 000-99 999	330 (40.7)	303 (40.9)	54 (17.8)	249 (82.2)
≥100 000	193 (23.7)	175 (23.6)	50 (28.6)	125 (71.4)
Total No. of medicines taken daily, mean (SD)	8.0 (4.4)	8.0 (4.4)	7.8 (4.6)	8.0 (4.3)

Abbreviation: SDM, shared decision-making.

^a^ Race was stratified based on whether patients identified as White or non-White.

A total of 151 clinicians (mean [SD] age, 45 [13.2] years; 75 [53.2%] were men, 111 [78.7%] were physicians, of which 103 [73.0%] were on staff) participated in these encounters: 76 (53.9%) of clinicians had worked 5 years or more in the current practice setting, half of them in either internal medicine or cardiology. Each evaluated a mean (SD) of 11.9 (19.6) patients per week for anticoagulation therapy, and 113 (88.3%) experienced burnout less than once a week (Table 2).

**Table 2.** Clinician Characteristics

Clinician characteristics	No. (%) (n = 151)^a^	Risk ratio (95% CI)^b^
Gender		
Male	75 (53.2)	1 [Reference]
Female	66 (46.8)	1.16 (1.02-1.33)
Age, mean (SD), y	45 (13.2)	NA
Per 10 y increase	NA	1.05 (0.99-1.11)
Physicians in residence		
Students or trainees^c^	38 (25.2)	1 [Reference]
In residence	113 (74.8)	1.24 (1.02-1.51)
Patients seen per week, mean (SD)	11.9 (19.6)	NA
Clinician type		
MD or DO	111 (73.5)	1 [Reference]
Other	40 (26.5)	1.03 (0.89-1.19)
Medical specialty setting		
Cardiology	34 (24.1)	1 [Reference]
Cardiac electrophysiology	27 (19.1)	1.05 (0.89- 1.24)
Family medicine	24 (17.0)	1.35 (1.15-1.58)
Internal medicine	35 (24.8)	1.23 (1.03-1.47)
Other	21 (14.9)	1.33 (1.18-1.51)
Years in practice		
≤4	65 (46.1)	1 [Reference]
>4	76 (53.9	1.03 (0.90-1.18)
Experience feelings of burnout		
Once a week or less	113 (88.3)	1 [Reference]
A few times a week or every day	15 (12)	1.16 (1.05-1.30)

Abbreviation: NA, not applicable.

^a^ A total of 141 clinicians completed the demographics survey.

^b^ Risk ratios (or relative risks) in this table refer to the ratio of the probability of a cost conversation occurring in a reference group to the probability of its occurrence in the comparator group (for example female vs male clinicians or family medicine vs cardiology).

^c^ This includes resident physicians, as well as nurses, physicians assistants, and pharmacy students.

Cost conversations occurred in 639 encounters, 378 (90.2%) in the SDM arm and 261 (63.5%) in the standard care arm. Cost conversations occurred in patients that had a mean (SD) age of 71.5 (10.4) vs 69.3 (10.4) years. Cost conversations were more prevalent among those with higher stroke risk (559 [78.3%] vs 80 [69.0%] with low stroke risk), and with middle-income patients (249 [82.2%] of those earning between $40 000 and $100 000 vs 203 [76.6%] of those earning less than $40 000 and 125 [71.4%] of those earning more than $100 000). In multivariable analysis, variables associated with cost conversations were patients in the SDM arm (OR, 9.69; 95% CI, 5.77-16.29), patients with a household income between $40 000 and $99 999 (OR, 1.86; 95% CI, 1.05-3.29) compared with an income of $100 000 or more, female clinicians (OR, 2.85; 95% CI, 1.21-6.71), clinicians practicing in family medicine (OR, 12.12; 95% CI, 2.75-53.38), internal medicine (OR, 3.82; 95% CI, 1.25-11.70) or other specialty (OR, 4.90; 95% CI, 1.32-18.16) when compared with cardiology, and staff clinicians vs those in training (OR, 4.01; 95% CI, 1.44-11.12). The estimates of effect size were not different in the per-protocol analysis (Table 3). Examples of the range of conversations that took place in these encounters is included in the eTable in Supplement 2.

**Table 3.** Multivariable Association of SDM Tool, Patient, Clinician, and Context Characteristics on Incidence of Cost Conversations

	Multivariable analysis, OR (95% CI)	
	Intention to treat analysis	SDM tool used
Use of SDM tool		
Arm (intervention arm)	9.69 (5.77-16.29)	NA
SDM tool (SDM tool used)	NA	11.73 (6.86-20.08)
Patient characteristics		
Age (per 10 y increase)	1.02 (0.99-1.04)	1.02 (0.99-1.04)
High stroke risk	1.00 (0.51-1.95)	1.05 (0.53-2.07)
Medication review cohort	0.84 (0.46-1.50)	0.83 (0.46-1.51)
Household income, $		
≥100 000	1 [Reference]	1 [Reference]
<40 000	1.36 (0.74-2.49)	1.38 (0.75-2.56)
40 000-99 000	1.86 (1.05-3.29)	1.82 (1.02-3.24)
Total No. of medicines taken daily (per 1 unit increase)	0.99 (0.94-1.05)	0.99 (0.94-1.04)
Clinician characteristics		
Women	2.85 (1.21-6.71)	2.73 (1.17-6.42)
Nonresident status	4.01 (1.44-11.12)	3.41 (1.21-9.61)
Non-MD or DO clinician	1.05 (0.37-2.96)	0.92 (0.33-2.58)
Medical specialty setting		
Cardiology	1 [Reference]	1 [Reference]
Cardiac electrophysiology	2.43 (0.85-7.00)	1.98 (0.69-5.68)
Family medicine	12.12 (2.75-53.38)	8.70 (2.00-37.86)
Internal medicine	3.82 (1.25-11.70)	2.91 (0.95-8.88)
Other	4.90 (1.32-18.16)	3.98 (1.08-14.73)

Abbreviations: OR, odds ratio; SDM, shared decision-making.

Patients who had cost conversations with their clinicians reported more frequently that cost influenced their decision of which anticoagulation agent to take when compared with patients who did not have cost conversations (patients reporting costs were one factor: OR, 3.66; 95% CI, 2.43-5.50; costs were sole factor: OR, 5.99; 95% CI, 1.85-19.37). However, the occurrence of cost conversations and the role of cost as a consideration was not associated with patient choice of medication during that visit (Table 4).

**Table 4.** Incidence of Cost Conversations and Cost as a Factor in Patient Decision-Making and Medication Choice

Survey response	Cost conversation, No. (%)		OR (95% CI)
	No (n = 191)	Yes (n = 639)	
**What choice did you make today for taking a blood thinner?**			
To take Warfarin	61 (20.3)	240 (79.7)	1 [Reference]
To take DOAC	94 (25.4)	276 (74.6)	0.75 (0.48-1.17)
Other decision	26 (20.6)	100 (79.4)	0.98 (0.56-1.71)
**Was the cost of the blood thinner a factor in your decision?**			
No, cost did not matter	147 (31.0)	327 (69.0)	1 [Reference]
Chose to take Warfarin	46 (28.9)	113 (71.1)	1 [Reference]
Chose to take DOAC	79 (31.3)	173 (68.7)	0.89 (0.56-1.42)
Chose other	22 (36.1)	39 (63.9)	0.72 (0.38-1.36)
Yes, cost was one factor I considered in my decision	30 (10.9)	244 (89.1)	3.66 (2.43-5.50)
Chose to take Warfarin	14 (12.5)	98 (87.5)	1 [Reference]
Chose to take DOAC	14 (13.0)	94 (87.0)	0.96 (0.51-1.80)
Chose other	2 (3.8)	52 (96.3)	3.71 (0.76-18.09)
Yes, cost was the sole factor in my decision	3 (7.0)	40 (93.0)	5.99 (1.85-19.37)
Chose to take Warfarin	1 (3.4)	28 (96.6)	1 [Reference]
Chose to take DOAC	1 (16.7)	5 (83.3)	0.18 (0.01-3.68)
Chose other	1 (12.5)	7 (87.5)	0.25 (0.01-4.49)

Abbreviations: DOAC, direct oral anticoagulants; OR, odds ratio.

## Discussion

Within a clinical trial using an SDM tool, cost conversations occurred in 3 of every 4 encounters between patients with AF and their clinicians. Use of an SDM tool and several clinician characteristics were significantly associated with cost conversations, whereas we could not find significant associations between patient characteristics other than income and the occurrence of cost conversations. Cost is influential in the choice of anticoagulant, but cost conversations were not associated with the selection of more or less expensive options.

Use of the SDM encounter tool was associated with a 10-fold increase in the odds of having a cost conversation during the encounter, a finding consistent with prior studies.^10,11^ While it is unclear how the Anticoagulation Choice SDM encounter tool may have supported cost conversations, it is possible that the cost content included in the tool, which highlighted the difference in out-of-pocket costs between warfarin and DOACs, helped at least identify cost as an important issue for patients with AF. It is also possible that the SDM tool prompted clinicians in general to more frequently ask about patient preferences, of which cost was one factor; patients using the tool also had more conversations about bleeding, anticoagulation treatment routine, reversing anticoagulation treatment, and diet/drug interactions.^19^ While the SDM tool was an important driver of cost conversations, we noticed that the incidence of cost conversations in the standard care arm (63%) was considerably higher than the incidence reported in other series^28,29^; clinician reports ranged from 15% to 50% of encounters, while patient reports ranged from 15% for newly prescribed medications to 44% for ongoing care visits. This higher-than-usual rate may reflect the decisional setting: anticoagulation to prevent AF-associated strokes involves considering potentially expensive alternatives and implementing treatments with substantial fidelity.

Some clinician characteristics were associated with cost conversations. Female clinicians were nearly 3 times more likely than their male counterparts to discuss costs. This may reflect patients’ expectations that female clinicians will engage in greater empathic communication and be more receptive to discuss biomedical and psychosocial issues in greater detail.^30^ Additionally, consultant clinicians were 4 times more likely to discuss costs than those in training, suggesting that clinician experience in managing AF includes bringing issues of cost to the conversation with patients. Furthermore, clinicians practicing family medicine, internal medicine, and other specialties were approximately 12, 4, and 5 times more likely than cardiologists to discuss costs, respectively. Although the reason for this finding is unclear, it may reflect differences in communication styles and attention to context between specialists and primary care clinicians.^31,32^ The only patient characteristic associated with having a cost conversation was income: patients with higher incomes (over $100 000) and lower incomes (less than $40 000) were less likely to have cost conversations relative to middle-income patients. These results reflect the association of financial capacity (for higher income) and the possible role of Medicare medication subsidies (for lower income) and the need to talk about cost of treatment. Taken together, these findings suggest that interventions to promote cost conversations may need to be preferentially directed at modifying clinician and practice factors rather than focus on patient-only interventions. This hypothesis should be explored further. In addition, more attention should be given to developing evidence-based interventions to support cost conversations, given that very few have been tested.^33^

Finally, we found that the occurrence of cost conversations was associated with patients who reported that cost was an important factor in their selection of anticoagulant. However, the actual choice of medication was not significantly correlated with the incidence of cost conversations, suggesting that either these conversations were not productive or consequential, or that considerations other than drug costs (eg, satisfaction with the existing regiment or the need to avoid diet restrictions or periodic monitoring) were more important in selecting an anticoagulation agent. Further analysis of the content and quality of these conversations will likely yield greater insight about their role in improving effective patient-centered care.

### Limitations

This study had several limitations. It is possible that the general nature of the costs offered in the SDM tool may have been sufficient to trigger cost conversations but in terms not individualized enough (ie, not able to estimate the exact out-of-pocket costs associated with each option) to support those conversations. Our findings may not apply broadly to the care of patients who would have opted not to participate in the SDM4AFib trial or to not have their encounter recorded, as happened with 10% of the trial’s encounters. Also, our results may not apply to other clinical contexts and health systems (eg, European). Our study also does not analyze conversation initiator, quality, length, and content (including direct vs indirect costs) of cost conversations; however, we provide a range of examples of conversations in the eTable in Supplement 2. In contrast to these limitations, our study draws from a rigorous and large randomized trial and is based on direct observations of clinical encounters in which actual treatment decisions were made, from which we made reproducible assessments of the occurrence of cost conversations.

## Conclusions

Cost conversations occurred in approximately 3 out of 4 encounters between patients with AF and their clinicians in this trial. These conversations are important as patients considered cost, and the patient’s own financial capacity to shoulder it, an important issue in the decision-making process. The use of an SDM tool, several clinician characteristics, and only 1 patient characteristic (income) were associated with the occurrence of a cost conversation. These findings can inform further efforts to promote helpful cost conversations in practice. With increasing costs of health care passed on to patients, these conversations are likely to be more widely relevant as patients co-create treatment programs with their clinicians and implement those programs in their lives with sufficient fidelity for them to be effective and safe.

## Notes

Supplement 1. Trial Protocol

Supplement 2. **eFigure.** Part of SDM Tool Highlighting Cost of Anticoagulation Options **eTable.** Examples of Conversation Content

Supplement 3. Nonauthor Collaborators
