# Supplementary material for: Cost Conversations About Anticoagulation Between Patients With Atrial Fibrillation and Their Clinicians: A Secondary Analysis of a Randomized Clinical Trial
Source: JAMA Netw Open. 2021 Jul 13;4(7):e2116009. doi: 10.1001/jamanetworkopen.2021.16009 (PMC8278261; doi:10.1001/jamanetworkopen.2021.16009)
Supplement: Supplement 1. — Trial Protocol [file jamanetwopen-e2116009-s001.pdf]

Shared decision making for stroke prevention in atrial fibrillation (SDM4Afib)

Investigators:
Peter Noseworthy, MD, Principal Investigator, Mayo Clinic
200 First Street SW, Rochester, MN 55905
(507) 293-0175
Noseworthy.peter@mayo.edu

Victor Montori, MD, Co-Investigator, Mayo Clinic (Rochester, MN)
Bruce Burnett, MD, Co-Investigator, Park Nicollet (St. Louis Park, MN)
Mark Linzer, MD, Co-Investigator, Hennepin Medical Center (Minneapolis, MN)
Elizabeth A. Jackson, MD, Co-Investigator, UAB Medicine - The University of Alabama at Birmingham (Birmingham, AL)
Erik P. Hess MD, Co-Investigator, UAB Medicine - The University of Alabama at Birmingham (Birmingham, AL)
James Hamilton, MD, Co-Investigator, University of Mississippi Medical Center (Jackson, MS)

Date of version: October 30, 2018
Version number: 1.8

Funding Source: National Heart, Lung, And Blood Institute of the National Institutes of Health under Award Number R01HL131535

Table of Contents

1.0 Aims ... 3
2.0 Background and Significance ... 3
3.0 Preliminary Work ... 5
4.0 Research Design & Methods ... 6
4.1 Schema ... 6
4.2 Study Setting and Participation ... 6
4.2.1 Eligibility Criteria for Clinicians ... 7
4.2.1.1 Enrollment of Clinicians ... 7
4.2.2 Eligibility Criteria for Patients ... 8
4.2.3 Identification of Subjects ... 9
4.3 Registration and Randomization of Patients ... 10
4.4 Intervention ... 11
4.4.1 Training of Clinicians ... **Error! Bookmark not defined.**
4.5 Standard Care ... 12
4.6 Data Collection ... 12
4.7 Outcome Measures ... 15
4.7.1 SDM quality ... 15
4.7.2 SDM processes ... 17
4.7.3 Anticoagulation Use ... 18
4.7.4 Safety outcomes ... 19
4.8 Follow-up Guidelines ... 19
4.9 Statistical Analysis ... 20
4.9.1 Analysis Plan ... 20
4.9.1.1 Missing Data ... 22
4.9.2 Sample Size Estimation ... 22
4.9.3 Site Allocation ... 23
5.0 Conflict of Interest ... 24
6.0 Human Subjects ... 24
6.1 Data safety monitoring board ... 24
6.2 Population ... 25
6.3 Potential Risks ... 25
6.4 Protection and Confidentiality ... 25
6.4.1 Subject privacy ... 25
6.4.2 Data management ... 26
6.4.3 Video and audio-recordings ... 26
6.4.4 Registry ... 26
6.4.5 IRB Umbrella ... 27
7.0 References ... 27

1.0 Aims

The goal of this study is to determine the extent to which standard care plus the Anticoagulation Choice tool promotes shared decision making (SDM) and impacts anticoagulation uptake and adherence versus standard care without this tool in patients with nonvalvular atrial fibrillation (AF).

Aim 1. To what extent does use of the ANTICOAGULATION CHOICE tool promote high-quality SDM versus standard care?

Using encounter video recordings and post-visit patient and clinician questionnaires, we will assess **SDM quality** (primary endpoint) **and processes.**

We hypothesize that use of the tool will improve SDM irrespective of patient literacy/numeracy, stroke risk, anticoagulation use at baseline, or type of clinic.

Aim 2. To describe the impact Anticoagulation Choice tool has on the rate of anticoagulation, the choice of anticoagulant, and adherence to anticoagulation in at-risk patients with AF versus the impact of standard care.

Using medical records and pharmacy profiles, we will determine the choice of anticoagulation, changes in anticoagulant use over time, and 12-month drug persistence, in all patients and in subgroups defined by patient literacy/numeracy, stroke risk, anticoagulation use at baseline, and type of clinic. As safety outcomes, we will monitor serious bleeding or strokes requiring medical attention.

2.0 Background and Significance

Atrial fibrillation is the most common cardiac arrhythmia affecting ~3 million Americans[1,2] It accounts for ~$26 billion/year in healthcare costs.[3] AF-related thromboembolic strokes are often devastating and a cause of great physical, social and economic burden.[4-7] Vitamin K

antagonists (VKAs, e.g., warfarin) reduce the risk of stroke by ~68%.[8-13] Recently, non-VKAs oral anticoagulants (NOACs) that directly inhibit factor Xa (e.g., rivaroxaban, apixaban, edoxaban) or thrombin (dabigatran) have demonstrated similar to or better efficacy and safety than warfarin.[14-16] Underuse of anticoagulation is a significant quality gap. Despite patients' strong aversion to strokes,[17,18] <50% of high-risk patients with AF receive anticoagulants.[19] Of these, 30-50% stop treatment within 12 months.[20-23] The low rate of anticoagulation suggests that clinicians are challenged in initiating anticoagulation, in part due to clinicians' aversion to causing anticoagulation-related bleeding,[19,24] Nonadherence suggests that some patients cannot implement anticoagulation in their lives: warfarin requires a stable diet and periodic laboratory (INR) monitoring,[25-27] while NOACs are costly and lack bleeding reversal agents.[14-16] Underuse may result also from poor patient and clinician access to, and deliberation with, individualized estimates of risks and benefits.[28,29] Patients and clinicians require support in initiating and implementing anticoagulation therapy.

In 2014, three major cardiovascular organizations formulated guidelines for the management of patients with AF. They gave their strongest class I recommendation for using SDM to individualize anticoagulation in at-risk AF patients.[30] SDM has the potential to support patients and clinicians in collaborative deliberation about reasonable anticoagulation strategies matched to medical risk and patient circumstance.[30-32] Nevertheless, this recommendation is based on expert consensus (level C evidence) and translating it into practice is challenging. The guideline provides no guidance on how to achieve this, and no tools were available that are both up-to-date and proven to support SDM in this context. Furthermore, we do not know what effect SDM may have on anticoagulation rates and adherence in patients with AF.[30]

We have developed and pilot tested a new online SDM tool (Anticoagulation Choice) to implement the 2014 class I recommendation in usual practice. The tool promotes a SDM conversation in the clinical encounter between the expert on important issues that bear on adherence, the patient, and the expert in medical issues, the clinician. Deliberating together on patient-important issues and medical matters, patients and clinicians can arrive at an evidence-based option that patients' value and can implement. Building on this experience,

we propose to implement SDM using the Anticoagulation Choice tool and evaluate its impact on SDM quality and on the rate by which patients take up anticoagulation and implement it in their lives.

3.0 Preliminary Work

The anticoagulation decision requires a conversation that discusses *both* the patient's risk of strokes and the issues that distinguish agents by fit with patient goals and situation. Using our user-centered design process, we created Anticoagulation Choice, a decision aid designed to support the recommendation for SDM for anticoagulation in AF. The development of the anticoagulation choice tool was built on 10 years of experience in designing decision aids that promote shared decision making and provide evidence-based content. The evidence-based content for this tool comes from systematic and expert reviews of randomized trials, observational studies, and qualitative studies.[30, 33-42] Simultaneously, we conducted 16 direct observations in primary and specialty clinics of clinical encounters in which anticoagulation decisions took place. The goal of these observations was to identify areas of opportunity to improve extant conversations.[43] The first "low-fidelity prototype"[44] was a rough-draft paper version and was field-tested within 8 clinical encounters. Iterations followed, first on paper, and then electronically, seeking to achieve patient engagement in the conversation. We judged this to have taken place when patients asked questions or made statements considering how anticoagulation would play out in their daily lives. An electronic version was necessary to ensure risk tailoring for each patient and to facilitate updating (we designed the tool to accommodate new evidence and new agents) and distribution. The online version supports conversations with patients who are new to anticoagulation as well as former and current warfarin users. Its use in field-testing required minimal support. The baseline risk, tailored to the patient using the CHA_2DS_2-VASc score (a tool that estimates risk of stroke), is shown using words, numbers, and a 100-person pictograph along with the expected risk reduction with anticoagulation. If this benefit is compelling to the patient consideration moves on to the salient issues differentiating the available options. The issue cards include the risk of bleeding (based on HASBLED, a tool that estimates risk of bleeding), availability of reversal agents, and practical considerations. Practical considerations include how each

choice affects patients' ability to be active, to travel, to eat a variety of meals, how the medicine is taken and its effects monitored, and what are the out-of-pocket costs. The final version of the tool is focused on the discussion of these issues after considering the risk of stroke and the risk reduction with anticoagulation. The tool is web-based and will be integrated where possible with the electronic workflow.

4.0 Research Design & Methods

We will conduct a multicenter randomized trial at the patient level comparing the Anticoagulation Choice tool and standard care versus standard care alone where enrolled clinicians will administer the intervention among patients with nonvalvular AF deemed at high risk of thromboembolic strokes. The study will assess the impact of the interventions on SDM quality and impact on anticoagulation use as well as monitoring safety concerns of strokes and bleeds. Also, as part of this trial, clinician training sessions will be evaluated to describe the normalization process of anticoagulation decision aid in the clinical sites. Data collection will include medical record review, survey completion, and note taking or video/audio recording of the clinical encounter and training sessions.

4.1 Schema

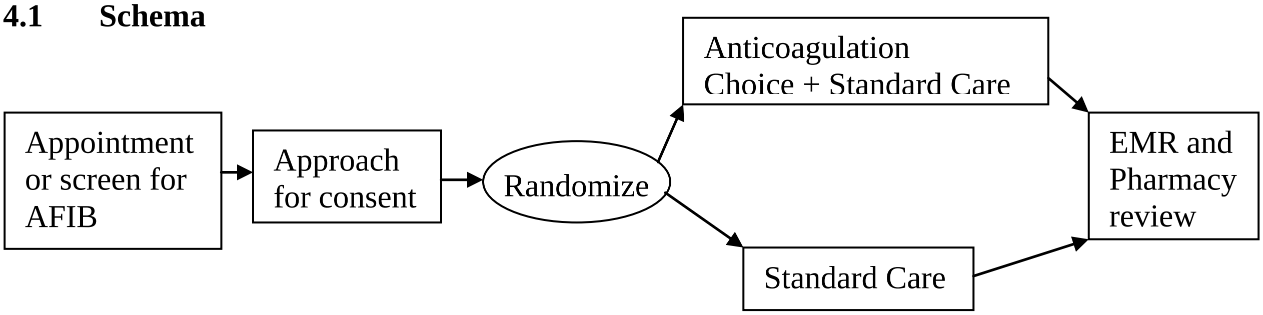

4.2 Study Setting and Participation

The trial will take place in clinics at Mayo Clinic (academic medical center), Park Nicollet Health Partners (urban/suburban community medical center), Hennepin County Medical Center (safety-net inner-city medical center), UAB Medicine - The University of Alabama at

Birmingham and University of Mississippi Medical Center that treat patients with atrial fibrillation.

At each recruiting location designated site staff will be trained to review informed consent documents and obtain necessary signatures from patients and clinicians and will be observed doing so, by study personnel or research staff, prior to actually consenting patients or clinicians.

4.2.1 Eligibility Criteria for Clinicians

All clinicians (MDs, NP/PAs, PharmDs) that are responsible for the modality of Anticoagulation in eligible AF patients at participating sites, without exclusion.

4.2.1.1 Enrollment of Clinicians

The research team and site champions will present an overview of the study at a department meeting. The informed consent document will be reviewed with interested clinicians before the clinician receives training on using the decision aid at the initial recruitment meeting or at their convenience throughout the duration of the study, prior to their first enrolled patient. Study staff will observe the clinician trainings, described in 4.2.1.2. The clinician will have the option to consent to recordings (video/audio or audio only) of clinical encounters with enrolled patients. If the clinician declines to do the recording they are still eligible for participation within the study. If the clinician agrees to recording of the clinical encounters on the consent they can still decline at time of the clinical encounter.

The study coordinator will quickly setup and start recording before leaving the room. The participants can stop this recording (video, aimed at the desk, or audio when the video camera is aimed at the ceiling) at any time (the device has a large red start/stop button and an on/off indicator light).

Consent only needs to occur one time (prior to being trained to use the decision aid and prior to the visit with the first enrolled patient). There will be no monetary or other sort of

reimbursement for clinicians participating in the trial. The participation of clinicians as subjects will not affect their current or future employment or be shared with their supervisor.

4.2.1.2 Training of Clinicians

Study personnel will do a demonstration in the use of the decision aid during in-person visits with participating clinics. Training session proceedings will be documented using discretionary video photographing, recording, or note-taking. Clinicians will complete a brief survey after trainings to describe promoting and inhibiting factors to the normalization of the anticoagulation decision aid in clinical practice. Similarly, transcripts and notes from trainings will undergo qualitative analysis to identify promoting and inhibiting factors to the implementation of the shared decision making tool in the clinical sites. Study personnel may also do a reminder of how to use the decision aid as needed (including just-in-time training) or in response to deviations in the quality of delivery observed on video/audio recordings. Brief video clips and storyboards that demonstrate the basic use of decision aids are publicly available at http://shareddecisions.mayoclinic.org for clinicians to review at their convenience.

4.2.2 Eligibility Criteria for Patients

Each criterion must be addressed and documented in the patient’s case report form for eligibility assessment by the study coordinator. No waivers or exemptions to any eligibility criteria will be permitted.

Inclusion Criteria:

1. ≥ 18 years of age
2. Nonvalvular AF deemed at high risk of thromboembolic strokes (CHA_2D_2-VASc Score ≥ 1 in men, or 2 in women).
3. Able to read and understand (despite cognitive, sensorial, hearing or language challenges) the informed consent document as determined by the study coordinator during consent.

Exclusion Criterion

1. Clinician indicates that patient is not a candidate for a discussion about anticoagulation medication.
2. Cognitive impairments
3. Mechanical values
4. Left appendage occlusion devices (example: Watchman)
5.

4.2.3 Identification of Subjects

Participants for all aims will be patients, their caregivers when pertinent, and clinicians. Participation is completely voluntary and we have procedures in place, sanctioned by the Mayo Clinic Institutional Review Board, Hennepin County Medical Center Institutional Review Board (HCMC), Park Nicollet Health Partners Institutional Review Board, UAB Medicine - The University of Alabama at Birmingham Institutional Review Board and University of Mississippi Medical Center Institutional Review Board to ensure that participants have the opportunity to opt out at any time and will not be further approached for participation or to provide data.

At the Mayo Clinic site, upcoming appointment lists for Atrial Fibrillation (AF) patients in primary care, cardiology, neurology, thrombophilia and anticoagulation clinics ECG result lists, medical records and clinician referrals will be reviewed for patient eligibility. Eligible patients will be approached and recruited in person, in a private location (i.e., clinic/exam room) prior to their appointment. Consent will occur by a trained research member as long as needed and until all questions by the subject have been answered. All study activities will occur within scheduled appointments, avoiding the need for additional research visits.

The patient and caregivers (if present), will be asked to provide consent to the recording (video/audio or audio only) of the clinical encounter. If the patient chooses to decline the recording they are still eligible to participate in the study. The study coordinator will quickly setup and start recording before leaving the room. The participants can stop this

recording (video, aimed at the desk, or audio when the video camera is aimed at the ceiling) at any time (the device has a large red start/stop button and an on/off indicator light).

The consent process will include the patient signing authorization to release protected health information forms to allow study personnel to obtain pharmacy prescription records and medical records from outside clinics. If a patient declines to sign an authorization form, he/she will still be eligible for the study but will be excluded from the analysis where information about medication and/or other medical records use is necessary (i.e. adherence analysis). The research team will contact the pharmacies and outside clinics for follow-up, so the patient will not be burdened with additional measures. There will be no monetary or other sort of reimbursement for participants.

4.3 Registration and Randomization of Patients

Prior to registering patients to the study, all of the eligibility criteria on the eligibility checklist will have been met.

Patients will be randomized by the study coordinator after completion of standard informed consent for participation in clinical research including permission to use protected health information.

Registration/randomization is available via REDCap (https://redcap1.mayo.edu/redcap/index.php), this is a secure, web-based application that is HIPPA compliant. Registration/randomization is available 24 hours a day via the REDCap website. Site staff will be provided a login and password by the study statistician.

Prior to accessing the REDCap website, site staff should verify the following:

- All eligibility criteria have been met.
- Informed consent has been obtained.
- Site staff has access to REDCap.

4.4 Intervention

In the intervention group, clinicians will conduct the encounter per standard care procedures with the addition of having access to the Anticoagulation Choice tool. The tool will be accessed online or through an available link in the Electronic Medical Record (EMR). Patient information to complete the calculators of risk (CHA_2DS_2-VASc) and bleeding (HAS-BLED; if needed) are: history of hypertension, congestive heart failure, stroke, vascular disease, diabetes mellitus, renal disease, liver disease, prior or predisposition to bleeding, unstable and/or high INR, whether the patient takes a medication predisposing him or her to bleeding, and the number of alcoholic drinks per week will be entered by the clinician into the tool or will be uploaded from the patients EMR to the tool and a personalized risk will be calculated (**Table 1**). CHA_2DS_2-VASc score of 0: recommend no antithrombotic therapy. CHA_2DS_2-VASc score of 1: recommend antithrombotic therapy with oral anticoagulation or antiplatelet therapy but preferably oral anticoagulation. CHA_2DS_2-VASc score ≥2: recommend oral anticoagulation.[2] A HAS-BLED score of ≥3 indicates that caution is warranted when prescribing oral anticoagulation and regular review is recommended.[2] Patients can request to receive a printed copy of the tool from their clinician which they can use later to share their decision with others, and to review, confirm or revisit their decision.

Table 1. Assessment of Stroke (CHA_2DS_2-VASc)[14] and Bleeding Risk (HAS-BLED)[15] in Atrial Fibrillation Patients

CHA_2DS_2-VASc	Score	HAS-BLED	Score
Congestive heart failure	1	Hypertension (SBP >160 mm Hg)	1
Hypertension	1	Abnormal renal and liver function[b]	1 or 2
Age ≥75 y	2	Stroke	1
Diabetes mellitus	1	Bleeding tendency/predisposition[c]	1
Stroke/TIA/TE	2	Labile INRs (if on warfarin)[d]	1
Vascular disease[a]	1	Elderly (e.g., age >65 y)	1
Aged 65 to 74 y	1	Drugs or alcohol (1 point each)[e]	1 or 2
Sex category (i.e., female sex)	1		

Maximum score	9	Maximum score	9

Acronym def.: TIA indicates transient ischemic attack; TE, thromboembolic; and INR, international normalized ratio.

a- Prior myocardial infarction (MI), peripheral artery disease (PAD), or aortic plaque.
b- Abnormal renal function is classified as the presence of chronic dialysis, renal transplantation, or serum creatinine ≥200 mmol/L. Abnormal liver function is defined as chronic hepatic disease (eg, cirrhosis) or biochemical evidence of significant hepatic derangement (bilirubin 2 to 3 times the upper limit of normal, in association with aspartate aminotransferase/alanine aminotransferase/alkaline. phosphatase 3 times the upper limit normal, etc). 1 point for each.
c- History of bleeding or predisposition (anemia).
d- Labile INR (ie, time in therapeutic range <60%).
e- Concomitant antiplatelets or nonsteroidal anti-inflammatory drugs, or excess alcohol.

4.5 Standard Care

The clinician will conduct the encounter per their standard of care. As access to the tool will be available to ensure contamination does not occur the study coordinator will inform the clinician prior to entering the room that the patient is to receive standard care and that the tool is not to be accessed.

4.6 Data Collection

Patients approached by study staff that agrees to participation will be captured in the remote data capture system (REDCap[45]). Potential eligible patients found to be ineligible or eligible but decline participation will be captured in a recruitment tracking log. The reason for ineligibility or reason for decline will be captured along with patients' age, sex, and race/ethnicity.

Self-reported responses from patients and clinicians will be collected at the end of the clinical encounter. At the time of their enrollment clinicians will complete a survey that collects data on their demographics. The post baseline survey will be given to the patient and clinician to complete at the clinic at the end of the encounter by the study coordinator or site appointed staff. Patients may be given the option to fill out part of the survey, prior to their visit if time allows. If a patient requests a return envelope, one will be provided to return the survey by mail. If the survey is not received in the 10 days post encounter a

reminder will be mailed to them with a copy of the survey along with a return envelope. A courtesy call will be made within 5 days post the mailing. Every effort will be made to complete the survey at the clinic immediately post encounter as this is the best chance for complete data collection. Another option for patients will be to have a follow up phone call approximately 1-2 days after their clinical encounter, to remind the patient to send their survey back or they will be given the option to complete their survey over the phone at that time.

Data from the medical record will be abstracted for all enrolled patients to capture demographic, clinical and medication prescription data. The time frame for collection will be from prior to enrollment to 12 months post enrollment. For patients that do not have any encounters at the institution for the past 12 months, a scan will be conducted up to 6 months after the 12-month timeline to verify continuity of care at the institution, change in contact information and/or survival status. If no records are available at that time, we will call the patient (number of attempts as authorized by each IRB), followed by a postal survey if nonresponse persist.

Data to be collected on patients include variables necessary to estimate the risk of stroke and bleeding, age, gender, BMI, smoking status, alcohol consumption, marital status, annual income of household, highest level of education, residency (nursing home), location of primary healthcare and total number of medications patients is currently taking. To further characterize the patients, we will use Chew et al single-item health literacy screener,[46] a 4-item modified Subjective Numeracy Scale,[47, 48] and a single-item health status measure.[49]

We will collect information on past use of anticoagulants through medical record review. We will categorize the patients into two cohorts for descriptive and analytical purposes. For patients who are not using an anticoagulant at the time of trial participation will form the ‘Start’ cohort. They may have used anticoagulation and discontinued >6 months ago, never used anticoagulation, or are using aspirin only. Patients that began an anticoagulant

within the past 10 days of the enrolled encounter that were prescribed an anticoagulant within the emergency department or an inpatient visit will still be considered a new ‘Start’.

Patients who are on warfarin or NOACs or used them in the past 6 months will form the ‘Review’ cohort. This cohort may include patients who have difficulty maintaining a therapeutic INR, or patients considering switching to a different anticoagulant or to stay on warfarin but switch to home INR monitoring.

The post consent survey for clinicians will collect demographic data (age, gender, specialty, % of their practice dedicated to anticoagulation care).

Calendar of Events

	**Prior to Study Enrollment**	**Prior to Encounter**	**RANDOMIZATION**	**Post Encounter**	**12 months Post Enrollment**
**Patient Completed Forms**					
Informed Consent	X				
Pharmacy Consent	X				
Survey				X	
Phone [1]					X
**Clinician Completed Forms**					
Informed Consent	X				
Survey		X		X	
**Clinical Data Abstracted from EMR**					
Bleeds					X
Strokes					X
INR Tests (# and values outside of 2-3 range)					X
Anticoagulation Prescription		X		X	X
**Pharmacist Request**					
Anticoagulation					X[2]

Use						

1- Patients who do not have utilization within enrolling healthcare system will be contacted via phone for verification of safety data (strokes and bleeds). If no information in the record and follow-up is necessary we will call patients the maximum number allowed by the IRB followed by a postal survey
2- Pharmacist records will be requested for 12 months prior to enrollment through 10 months post enrollment.

4.7 Outcome Measures

4.7.1 SDM quality

SDM quality will measure (a) knowledge transfer; (b) concordance; (c) quality of communication and satisfaction with shared decision making; and (d) satisfaction with the decision-making process.

Knowledge transfer is 6 questions about AF and anticoagulation. The 6 questions use a response format "true / false / do not know", and are answered with full access to the decision aids since they are not meant to test recall. Correct responses will be summed and divided by the total number of questions asked. If a patient answers at least 1 knowledge question then they will be assessed for this outcome, where all missing responses will be coded as incorrect.

Knowledge of risk will contain one question that asks patients to estimate their own risk of stroke. Correct answers will be within ± 10% (strict score) and ± 30% (liberal score) *relative* to the calculated risk estimate.

Collaborative agreement will assess decision concordance between the patient and the clinician. Both the patient and clinician will be asked to report about what decision (anticoagulation no/yes-which one) was made during the index visit. Agreement will be calculated between both parties and reported.

Patient decision satisfaction will be assessed using the Decisional Conflict Scale (DCS).[50, 51] The 16 items of DCS are scored on a 0-4 scale; the items are summed, divided by 16 and

then multiplied by 25. The scale is from 0-100 where higher scores are reflective of uncertainty about the choice. There are 5 DCS subscales, where a DCS subscale consists of 3 questions (1 subscale of 4). If 2 of 3 (or 3 of the 4) questions within a subscale have responses, then the patient would be considered as a responder and a score could be calculated. If more than one response per subscale is missing then that specific subscale is not calculated for the patient. An overall DCS score can be calculated if no more than 5 responses are missing as long as each missing response falls into a different subscale.

Quality of Communication will be assessed with a modified version of three questions from the CAHPS Clinician and Group survey[52]. CAHPS surveys include questions to assess patient perspectives of communication with their clinician. These questions indicate the extent to which the communication is patient-centered. Three questions ask about specific aspects of technical (explain things in a way you could understand) and affective (show respect for what you have to say) communication. Each item is assessed on a 3 point scale (Yes, definitely; Yes, somewhat and No) that will be individually reported, no composite score will be done. Three modifications are made to improve the relevance of the items to the present study: (1) Instructions were changed from "These questions ask about your most recent visit with this doctor. Please answer only for your own health care." to "Thinking of the conversation you just had with your clinician about blood thinners (anticoagulation medications), please select the most appropriate response to each item below." (2) "During your most recent visit" was removed from the item stems. (3) "This doctor" was replaced with "this clinician."

Patient satisfaction with encounter will be assessed with 1 question on a 7 point likert scale. Patients will be asked whether they would recommend the approach used to others for other discussions.

Clinician satisfaction with encounter will be assessed with 2 questions. A 5 point likert scale questioning satisfaction with discussion about anticoagulation medication choice. The clinician will also be asked whether they would recommend the approach used to other clinicians for other discussions on a 7 point Likert scale.

4.7.2 SDM processes

To assess SDM processes the recordings of the clinical encounter will be evaluated (video/audio or audio only recordings).

Extent of SDM that took place during the encounter will be assessing the degree of involvement of patients by the clinician in SDM using the OPTIONS scale.[53] The scale consists of 12 items scored from 0, no effort to 4, exemplary effort. The 12 items are summed and converted to a 100 point scale. A sample of 20% of the recordings will be assessed by two or more reviewers. Agreement will be assessed by Lin's concordance index[54], where any value over 0.8 will be considered concordant. If concordance does not occur within the first 20%, the two reviewers will assess cases of difference and review an additional 10 cases to test for agreement. Recordings scored by both reviewers will be averaged.

Impact of SDM on Encounter will be assessed by comparing the length in minutes of the discussion about anticoagulation and of the office visit, when available. Study coordinators when possible will time the encounters in intervention and control visits, prioritizing those encounters in which recording was not allowed. Potential issues preventing assessment of time may be recruitment of another patient.

Fidelity of SDM Tool by the clinician will be assessed by a review of the recording looking for key items to be addressed. A checklist of key elements will be assessed in both arms to assess not only the fidelity but potential contamination. A sum of the components in the checklist will be calculated for each recording and compared between arms. To score the recording first a sample of 20% of the video's will be assessed by two reviewers. Agreement will be assessed by Lin's concordance index[54], where any value over 0.8 will be considered concordant. If concordance does not occur within the first 20%, the two reviewers will assess cases of difference and review an additional 10 cases to test for agreement. Recordings scored by both reviewers will be averaged. For encounters where

audio and video recording has been declined, a real-time assessment will occur at the consent of the clinician and the patient. The study coordinator will conduct these real-time reviews.

Inclusion of Cost as an Element of SDM Process will be assessed by first using qualitative inductive content analysis of the transcripts of video-recorded clinical encounters to describe the scope of cost conversations. Deductive video-graphic analyses will be used to code the occurrence of cost conversation themes in order to determine the impact that Anticoagulation Choice has on the appearance of these themes, controlling for individual characteristics and contexts, and the association between cost conversation themes and SDM quality (described above), SDM Processes (described above), and Anticoagulation Use (described below).

4.7.3 Anticoagulation Use

Rate of anticoagulation: The key indicator of the choice to start an anticoagulant will be its prescription in the EMR prescription module (observed discussions and patient/clinician accounts may not reflect decisions confirmed after the visit with a prescription, for example, after the clinician or the patient checked other information or with other informants). After this primary 'decided as prescribed' approach, we will conduct secondary analyses using patient/clinician reported and video-observed decisions. Decisions may be for starting or not an anticoagulant in the start cohort. It is possible that there may be some decisions to stop anticoagulation in the review cohort, but we expect start and review cohorts to contribute information about choice of anticoagulant.

Choice of anticoagulant**:** We will review the EMR and 10-month pharmacy profiles (to stand for the 12-month profile given the automatic expiration of pharmacy records at 1 year) to determine the prescribed anticoagulant and whether and when switches to another agent or to no anticoagulant took place.

Together, they should capture choice and switches even when these occur as a result of changes in clinician (e.g., from cardiology to primary care). When available, we will note the documented reasons from clinical notes for choosing and switching as well as with which clinician the change was made (e.g primary, cardiology, etc.).

Anticoagulation persistence: Patients will identify the pharmacy(-ies) they use to fill their prescriptions and authorize us to obtain their prescription drug fill data. We will calculate anticoagulation persistence, using the percent days covered (PDC) based on prescription refill behaviors (total days supply of anticoagulant filled / total days of observation from the first prescription fill date; range 0-100%). We will also pull all pharmacy refills for the 12 months prior to enrollment. This will allow us to calculate persistence for prior use of anticoagulants for the review cohort to compare to persistence post encounter and see if there is an impact.

Warfarin use: For patients who choose to stay on warfarin, we will also use as secondary measures of adherence: (a) the proportion of INR tests obtained/scheduled; and (b) percentage of time at therapeutic target (typically INR 2-3).

4.7.4 Safety outcomes

Strokes and bleeds requiring medical assistance will be monitored and reported to the data safety monitoring board (section 6.1). Because very few of these are expected, we will rely on patient/clinician self-report and medical record review 12 months post enrollment for each participant. Should a patient not have utilization in the 3 months prior to the 12 month date, then the patient will be contacted directly for confirmation.

4.8 Follow-up Guidelines

All patients will be followed per protocol guidelines and deviations from protocol will be reported to the IRB.

<u>Withdraw</u>: If a patient refuses to continue to participate and they withdraw consent they will then be considered withdrawn from the study; to uphold the intention-to-treat principle, we will inquire as to whether we can continue to passively collect data from the medical record, and if ok from 10-12 month pharmacy profiles and patient surveys. If not, then no further data will be collected through medical review or self-report. Data collected prior to withdraw will be utilized unless expressly told otherwise by the patient.

<u>Ineligible</u>: If a patient enrolled onto the study has been found ineligible (not meeting one of the eligibility criteria) they will be documented for reason of ineligible in the study chart. These patients will continue to receive the intervention and all data will be collected for the study. This is a safe course of action as the intervention does not pose any potential harm to the patient beyond loss of privacy. The patients will be identified in the results as being ineligible and reason but will be included in all analyses.

<u>The procedure for post-randomization exclusions will involve presenting the case to the trial PI blind to the participant's allocation and to their results.</u>

4.9 Statistical Analysis

4.9.1 Analysis Plan

The study will be analyzed according to the *intention to treat principle (ITT)*, including all patients enrolled to the study in the arm to which they were assigned, regardless of which they were assigned to (e.g., standard care or Anticoagulation Choice + Standard Care). Reporting will include 'Per Protocol', complete data for each arm plus the ITT analysis where imputation analysis methods will be utilized to address any missing values (see section 4.9.1.1 for details on missing data analysis). Baseline characteristics will be reported in the study results with continuous values being reported as means and standard deviations and categorical values reported as counts and frequencies and compared between study arms using t-tests and chi-squared tests. Any baseline imbalances ($p<0.05$) will be will be explored as a possible factor to adjust for when the outcome measures are

analyzed. We will adhere to the CONSORT guidelines to transparently report study results and ensure that sufficient information is included to allow for assessment of the study's internal and external validity.

We will use standard techniques appropriate for trials, with each outcome compared between study arms using t-tests for continuous outcomes and chi-square tests for dichotomous outcomes. If there are differences in baseline characteristics found by statistical means or found to have clinical relevance between the two study groups, these will be accounted for using regression models which include an indicator for study arm.

We will perform descriptive analyses to describe any potential heterogeneity of treatment effect (HTE) and facilitate synthesis of subgroup results in future meta-analyses. We will conduct descriptive HTE analyses by clinic (academic, community and safety net), by cohort (start or review cohort), by stroke risk (CHA2DS2-VASc score of 1 or ≥ 2 for men and 2 or ≥ 3 for women), and by numeracy (Less than adequate vs. not). The outcomes assessed with HTE analyses will be the same as those assessed in the trial (e.g., SDM and communication quality, knowledge, and decisional satisfaction).

For the main analyses (SDM Quality and SDM Processes), we will not assume that patient effectiveness outcomes are independent of the clinician, but rather test to see if patients seen by the same clinician have correlated outcomes. Ignoring such "clustering" effects would result in over-narrow confidence intervals and potentially false positive study results. Instead, if clustering is seen, determined by calculating the intra-class correlation (ICC > 0.05) for each outcome, then the value for the ICC will be reported in findings. We will use cluster (cluster at clinician level) adjusted t-test and chi-square test for comparisons between arms and hierarchical generalized linear models (HGLMs) with random main effects specified at the clinician level when adjusting by more than arm.[55] If clustering is not present then the results will reduce to a model that assumes independence and reflect findings appropriately.

Some data analysis will be conducted at the Leiden University Medical Center (the Netherlands), by using remote access connection to the Mayo server and during the appointment time on Mayo Clinic campus. All data will be stored securely on a password-protected computer. Password-protected USB drives may also be used to store electronic files in situations where connection to the Mayo server is limited or unavailable. These USB drives are encrypted and will be used in accordance with Mayo Clinic's Portable Computing and Telecommunication Devices Policy. These USB drives may be shared externally to the Leiden University Medical Center (the Netherlands).

4.9.1.1 Missing Data

We will make every effort to minimize missing data. Trial enrollment and the fidelity of follow-up procedures will be reviewed during bi-weekly conference calls. A study biostatistician will conduct frequency reports to assess for missing data, and the study team, which is experienced in conducting multicenter trials, will trouble shoot any problems encountered. We will report rates of missing data for each outcome by study arm and send missing data reports to sites.

4.9.2 Sample Size Estimation

The table shows the detectable effect for each of the outcomes of interest if we were to have data on that outcome from a total of 333 patients (1% of available population). This provides enough power ($\alpha=0.05$; two-sided difference) to detect meaningful differences across arms for all SDM quality and process outcomes. Our intent, however, is to have enough power to detect important differences when we conduct analyses of groups or cohorts of patients. Most of these analyses will divide the participants into 2 cohorts (e.g., start and review cohorts), except for the subgroup analysis by clinic, which divides the total sample into 3 cohorts: academic, community, and safety net clinic. That is the only analysis with three groups. Given this, we would need 3 times the sample size listed in the table, or **999 participants (3% of available sample)** to address all planned subgroup analyses. These are minimum targets for recruitment and we do <u>not</u> plan to limit recruitment in any way to enroll up

to this number of patients. It assumes even distribution of participants per subgroup (e.g., start vs. review cohort); since the only grouping with three levels is clinic, each clinic will be expected to recruit similar numbers. Thus, for the main analysis and for other subgroup analyses (n=2 levels),

**Outcome (n = 333)**	**Rate (%) or SD**	**Detectable effect**	**Power***
**Patient level – SDM quality**			
Knowledge transfer^	18	5.6	84%
Knowledge of risk	55%	15%	81%
Decisional conflict scale^	17	5.2	80%
**Clinician level**			
Satisfaction^	54%	15%	80%
**Encounter level – SDM process**			
Engagement (OPTION12)^	12.6	3.9	80%

^ Values from iADAPT SDM tool trial; * α=0.05; two-sided

We expect approximately 90% of patients to start (start cohort) or continue a medication (review cohort). Of those, we can reasonably expect to obtain >85% of the pharmaceutical records for those (records will be requested of all enrolled patients regardless of decision). Thus, using the trial size estimated of **999 participants**, we will have ~765 patient records available for assessment of anticoagulant persistence at 12 months (PDC). In our review of the Optum database, 40% of patients were adherent to anticoagulation (>80% PDC, the threshold used by CMS) at 12 months. Assuming an expected rate of 60% PDC for the usual care cohort, we would have 80% power to detect a 9% difference (69% PDC in the SDM tool arm), with a two-sided test and an alpha of 0.05. In subgroup analyses comprising 100 participants per arm and using a one-sided test and alpha of 0.05, we will have 80% power to detect differences of at least 16%.

4.9.3 Patient Allocation

Eligible patients will be allocated into either the usual care arm or to the usual care + ANTICOAGULATION CHOICE SDM tool (intervention) arm using a random sequence the trial statistician will generate *a priori*. The allocation will be stratified by clinic (academic, community or safety net), by cohort (start or review), and stroke risk (CHA_2DS_2-VASc score of 1 or ≥ 2 for men and 2 or ≥ 3 for women) using blocks of random size.

5.0 Conflict of Interest

The tool under evaluation is not part of any existing effort to commercialize or profit from its use; the researchers involved in this study have not received -- and will not receive with their application in this study -- any royalties or other monetary benefits, directly or indirectly, from the use of the decision aids or from the makers of the interventions being discussed in this tool.

6.0 Human Subjects

6.1 Data safety monitoring board

A Data Safety and Monitoring Plan (DSMP) and charter has been formed to monitor participant safety, data completeness and adherence to study protocol. In addition, the principal investigator, each of the site investigators and champions, study statisticians, and project coordinator will meet monthly to assess recruitment (overall and by site), baseline comparability of treatment groups, protocol adherence, completeness of data collection, safety, and fidelity of follow-up procedures. They will meet monthly or as needed to review safety. Any potential adverse events will be entered into the study database and the Institutional Review Board will be notified. A Data Safety Monitoring Board (DSMB) has been formed and will meet bi-annually or as needed starting just prior to study enrollment.

6.2 Population

This study will be available to all eligible patients, regardless of race, gender, or ethnic origin. There is no information currently available regarding differential outcomes of the decision aid in subsets defined by race, gender, or ethnicity, and there is no reason to expect such differences to exist.

6.3 Potential Risks

Potential risks to patient subjects should be minimal. Given that the intervention has been extensively pilot tested and no adverse outcomes occurred, we do not expect early termination due to harm. The intervention is an educational tool for use during the clinic visit to help patients make decisions about anticoagulation medications. The tool does not make recommendations or result in prescriptions without the participation of the clinician, and the tool is not to be used outside a clinical visit in which a clinician can place the information in context.

6.4 Protection and Confidentiality

6.4.1 Subject privacy

In this study, the privacy of all study participants will be protected by avoiding the use of names on all research data (including field notes, transcribed conference call, meeting tapes, audio-and video-recordings of the interviews, transcripts). All study participants will be identified by a unique study code number only. The link between the code number and study participant's identity will be stored within the remote data capture system. Medical records will be abstracted electronically using computers that are not linked to any Mayo mainframe computer. Names of those who decline participation in the study will be maintained in a do-not-contact list, so they will not be contacted multiple times (as patients are likely to have multiple appointments at participating clinics during the study). All research material outside of what is stored within the remote data capture system will be maintained on a secure server at the KER Unit at Mayo Clinic and locked in file cabinets.

6.4.2 Data management

All sites will be required to use the current version of all documents and forms and adhere to the study schedule. Forms and documents will be returned to study coordinators via upload to the remote data entry system (REDCap[44]), Fedex or data transfer between sites. Data entry specialists will enter survey and medical record data into the REDCap system which is hosted by Mayo Clinic, which is a HIPAA compliant secure data entry system that allows for validated data entry, edit checks and logs of all data changes. The data can be accessed by the statistical team at any time and downloaded into a statistical software package. The statistical team will review the data on a bi-weekly basis to ensure data accuracy and completeness. All data, documents, and analysis findings will be housed within the Mayo Clinic system that is password protected and backed up on a nightly basis. The data will be stored within the secure system following completion of the study.

6.4.3 Video and audio-recordings

Health visits will be recorded (video/audio or audio only) with permission of all participants. These recordings are conducted using a portable digital video camera that is placed aimed at the clinician’s desk, away from the physical examination table. The clinician and the patient will be instructed on how to occlude the lens, direct the camera to a wall, or turn off the camera at any time they feel this is appropriate. Digital recordings are immediately transferred and/or uploaded to the research team’s secure server and deleted from portable devices after overnight back-up of Mayo’s servers. The video and audio files are identified using a code number that does not include the name of the clinician, support staff, or patient or reference to their medical record number or date of birth. All recordings will be transcribed verbatim by an IRB approved transcription service.

6.4.4 Registry

Collected study data (including audio and video recordings) will be kept in a registry to conduct further analyses, future un-identified and IRB approved research, trainings, quality

improvement and educational purposes, which includes sending data (and recordings) to research collaborators. The research collaborators will have research appointments with Mayo Clinic and will only assist in analyzing data; they will not have contact with study participants.

6.4.5 IRB Umbrella

When the study is being kept open for secondary analysis and registry purposes only, this IRB application may merge to an umbrella IRB application.

7.0 References

1. Lloyd-Jones DM, Wang TJ, Leip EP, et al. Lifetime risk for development of atrial fibrillation: the Framingham Heart Study. *Circulation.* 2004;110(9):1042-1046.
2. Naccarelli GV, Varker H, Lin J, Schulman KL. Increasing prevalence of atrial fibrillation and flutter in the United States. *Am J Cardiol.* 2009;104(11):1534-1539.
3. Kim MH, Johnston SS, Chu BC, Dalal MR, Schulman KL. Estimation of total incremental health care costs in patients with atrial fibrillation in the United States. *Circ Cardiovasc Qual Outcomes.* 2011;4(3):313-320.
4. Lin HJ, Wolf PA, Kelly-Hayes M, et al. Stroke severity in atrial fibrillation. The Framingham Study. *Stroke.* 1996;27(10):1760-1764.
5. Lamassa M, Di Carlo A, Pracucci G, et al. Characteristics, outcome, and care of stroke associated with atrial fibrillation in Europe: data from a multicenter multinational hospital-based registry (The European Community Stroke Project). *Stroke.* 2001;32(2):392-398.
6. Feigin VL, Forouzanfar MH, Krishnamurthi R, et al. Global and regional burden of stroke during 1990- 2010: findings from the Global Burden of Disease Study 2010. *Lancet.* 2014;383(9913):245-254.
7. Go AS, Mozaffarian D, Roger VL, et al. Heart disease and stroke statistics--2014 update: a report from the American Heart Association. *Circulation.* 2014;129(3):e28-e292.
8. Petersen P, Boysen G, Godtfredsen J, Andersen ED, Andersen B. Placebo-controlled, randomised trial of warfarin and aspirin for prevention of thromboembolic complications in chronic atrial fibrillation. The Copenhagen AFASAK study. *Lancet.* 1989;1(8631):175-179.
9. Ezekowitz MD, Bridgers SL, James KE, et al. Warfarin in the prevention of stroke associated with nonrheumatic atrial fibrillation. Veterans Affairs Stroke Prevention in Nonrheumatic Atrial Fibrillation Investigators. *N Engl J Med.* 1992;327(20):1406-1412.

10. Stroke Prevention in Atrial Fibrillation Study. Final results. *Circulation.* 1991;84(2):527-539.
11. Warfarin versus aspirin for prevention of thromboembolism in atrial fibrillation: Stroke Prevention in Atrial Fibrillation II Study. *Lancet.* 1994;343(8899):687-691.
12. The effect of low-dose warfarin on the risk of stroke in patients with nonrheumatic atrial fibrillation. The Boston Area Anticoagulation Trial for Atrial Fibrillation Investigators. *N Engl J Med.* 1990;323(22):1505-1511.
13. Connolly SJ, Laupacis A, Gent M, Roberts RS, Cairns JA, Joyner C. Canadian Atrial Fibrillation Anticoagulation (CAFA) Study. *J Am Coll Cardiol.* 1991;18(2):349-355.
14. Connolly SJ, Ezekowitz MD, Yusuf S, et al. Dabigatran versus warfarin in patients with atrial fibrillation. *N Engl J Med.* 2009;361(12):1139-1151.
15. Patel MR, Mahaffey KW, Garg J, et al. Rivaroxaban versus warfarin in nonvalvular atrial fibrillation. *N Engl J Med.* 2011;365(10):883-891.
16. Granger CB, Alexander JH, McMurray JJ, et al. Apixaban versus warfarin in patients with atrial fibrillation. *N Engl J Med.* 2011;365(11):981-992.
17. Haynes RB, Devereaux PJ, Guyatt GH. Clinical expertise in the era of evidence-based medicine andpatient choice. *Vox Sang.* 2002;83 Suppl 1:383-386.
18. Lahaye S, Regpala S, Lacombe S, et al. Evaluation of patients' attitudes towards stroke prevention andbleeding risk in atrial fibrillation. *Thromb Haemost.* 2014;111(3):465-473.
19. Ogilvie IM, Newton N, Welner SA, Cowell W, Lip GY. Underuse of oral anticoagulants in atrial fibrillation: a systematic review. *Am J Med.* 2010;123(7):638-645 e634.
20. Gallagher AM, Rietbrock S, Plumb J, van Staa TP. Initiation and persistence of warfarin or aspirin in patients with chronic atrial fibrillation in general practice: do the appropriate patients receive stroke prophylaxis? *J Thromb Haemost.* 2008;6(9):1500-1506.
21. Hylek EM, Evans-Molina C, Shea C, Henault LE, Regan S. Major hemorrhage and tolerability of warfarin in the first year of therapy among elderly patients with atrial fibrillation. *Circulation.* 2007;115(21):2689-2696.
22. Mant J, Hobbs FD, Fletcher K, et al. Warfarin versus aspirin for stroke prevention in an elderly community population with atrial fibrillation (the Birmingham Atrial Fibrillation Treatment of the Aged Study, BAFTA): a randomised controlled trial. *Lancet.* 2007;370(9586):493-503.
23. Rash A, Downes T, Portner R, Yeo WW, Morgan N, Channer KS. A randomised controlled trial of warfarin versus aspirin for stroke prevention in octogenarians with atrial fibrillation (WASPO). *Age Ageing.* 2007;36(2):151-156.
24. Glazer NL, Dublin S, Smith NL, et al. Newly detected atrial fibrillation and compliance with antithrombotic guidelines. *Arch Intern Med.* 2007;167(3):246-252.
25. Platt AB, Localio AR, Brensinger CM, et al. Can we predict daily adherence to warfarin?: Results from the International Normalized Ratio Adherence and Genetics (IN-RANGE) Study. *Chest.* 2010;137(4):883-889.

26. Couris R, Tataronis G, McCloskey W, et al. Dietary vitamin K variability affects International Normalized Ratio (INR) coagulation indices. *Int J Vitam Nutr Res.* 2006;76(2):65-74.
27. Franco V, Polanczyk CA, Clausell N, Rohde LE. Role of dietary vitamin K intake in chronic oral anticoagulation: prospective evidence from observational and randomized protocols. *Am J Med.* 2004;116(10):651-656.
28. Seaburg L, Hess EP, Coylewright M, Ting HH, McLeod CJ, Montori VM. Shared decision making in atrial fibrillation: where we are and where we should be going. *Circulation.* 2014;129(6):704-710.
29. Devereaux PJ, Anderson DR, Gardner MJ, et al. Differences between perspectives of physicians and patients on anticoagulation in patients with atrial fibrillation: observational study. *Bmj.* 2001;323(7323):1218-1222.
30. January CT, Wann LS, Alpert JS, et al. 2014 AHA/ACC/HRS guideline for the management of patients with atrial fibrillation: a report of the American College of Cardiology/American Heart Association Task Force on Practice Guidelines and the Heart Rhythm Society. *J Am Coll Cardiol.* 2014;64(21):e1-76.
31. Charles C, Gafni A, Whelan T. Decision-making in the physician-patient encounter: revisiting the shared treatment decision-making model. *Soc Sci Med.* 1999;49(5):651-661.
32. Montori VM, Gafni A, Charles C. A shared treatment decision-making approach between patients with chronic conditions and their clinicians: the case of diabetes. *Health Expect.* 2006;9(1):25-36.
33. McGrath ER, Kapral MK, Fang J, et al. Association of atrial fibrillation with mortality and disability after ischemic stroke. *Neurology.* 2013;81(9):825-832.
34. Clarkesmith DE, Pattison HM, Lane DA. Educational and behavioural interventions for anticoagulant therapy in patients with atrial fibrillation. *Cochrane Database Syst Rev.* 2013;6:CD008600.
35. Gomez-Outes A, Terleira-Fernandez AI, Calvo-Rojas G, Suarez-Gea ML, Vargas-Castrillon E. Dabigatran, Rivaroxaban, or Apixaban versus Warfarin in Patients with Nonvalvular Atrial Fibrillation: A Systematic Review and Meta-Analysis of Subgroups. *Thrombosis.* 2013;2013:640723.
36. Salazar CA, del Aguila D, Cordova EG. Direct thrombin inhibitors versus vitamin K antagonists for preventing cerebral or systemic embolism in people with non-valvular atrial fibrillation. *Cochrane Database Syst Rev.* 2014;3:CD009893.
37. Lapner S, Cohen N, Kearon C. Influence of sex on risk of bleeding in anticoagulated patients: a systematic review and meta-analysis. *J Thromb Haemost.* 2014;12(5):595-605.
38. Biondi-Zoccai G, Malavasi V, D'Ascenzo F, et al. Comparative effectiveness of novel oral anticoagulants for atrial fibrillation: evidence from pair-wise and warfarin-controlled network metaanalyses. *HSR Proc Intensive Care Cardiovasc Anesth.* 2013;5(1):40-54.
39. Verdecchia P, Angeli F, Bartolini C, et al. Safety and efficacy of non-vitamin K oral anticoagulants in non-valvular atrial fibrillation: a Bayesian meta-analysis approach. *Expert Opin Drug Saf.* 2015;14(1):7-20.

40. Limone BL, Baker WL, Kluger J, Coleman CI. Novel anticoagulants for stroke prevention in atrial fibrillation: a systematic review of cost-effectiveness models. *PLoS One.* 2013;8(4):e62183.
41. Zhu WG, Xiong QM, Hong K. Meta-Analysis of CHADS versus CHADS-VASc for Predicting Stroke and Thromboembolism in Atrial Fibrillation Patients Independent of Anticoagulation. *Tex Heart Inst J.* 2015;42(1):6-15.
42. LeBlanc A, Bodde AE, Branda ME, et al. Translating comparative effectiveness of depression medications into practice by comparing the depression medication choice decision aid to usual care: study protocol for a randomized controlled trial. *Trials.* 2013;14:127.
43. Rudd J, Stern K, Isensee S. Low vs. high-fidelity prototyping debate. *interactions.* 1996;3(1):76-85.
44. Paul A. Harris, Robert Taylor, Robert Thielke, Jonathon Payne, Nathaniel Gonzalez, Jose G. Conde, Research electronic data capture (REDCap) - A metadata-driven methodology and workflow process for providing translational research informatics support, J Biomed Inform. 2009 Apr;42(2):377-81.
45. Chew LD, Bradley KA, Boyko EJ. Brief questions to identify patients with inadequate health literacy. *Family medicine.* 2004;36(8):588-594.
46. Chew LD, Griffin JM, Partin MR, et al. Validation of screening questions for limited health literacy in a large VA outpatient population. *J Gen Intern Med.* 2008;23(5):561-566.
47. Fagerlin A, Zikmund-Fisher BJ, Ubel PA, Jankovic A, Derry HA, Smith DM. Measuring numeracy without a math test: development of the Subjective Numeracy Scale. *Med Decis Making.* 2007;27(5):672-680.
48. Zikmund-Fisher BJ, Smith DM, Ubel PA, Fagerlin A. Validation of the Subjective Numeracy Scale: effects of low numeracy on comprehension of risk communications and utility elicitations. *Med Decis Making.* 2007;27(5):663-671.
49. Cunny KA, Perri M, 3rd. Single-item vs multiple-item measures of health-related quality of life. *Psychological reports.* 1991;69(1):127-130.
50. Stacey D, Legare F, Col NF, et al. Decision aids for people facing health treatment or screening decisions. *The Cochrane database of systematic reviews.* 2014;1:CD001431.
51. O'Connor AM. Validation of a decisional conflict scale. *Med Decis Making.* 1995;15(1):25-30.
52. CAHPS (https://cahps.ahrq.gov/clinician_group)
53. Barr PJ, O'Malley AJ, Tsulukidze M, Gionfriddo MR, Montori V, Elwyn G. The psychometric properties of Observer OPTION, an observer measure of shared decision making. *Patient Educ Couns.* 2015.
54. Lawrence I-Kuei Lin. A Concordance Correlation Coefficient to Evaluate Reproducibility. *Biometrics* 45.1 (1989): 255-68.
55. Donner A, Klar N. Design and analysis of cluster randomized trials in health research. London: Arnold Publishers; 2000.
