# Supplementary material for: Cost Conversations About Anticoagulation Between Patients With Atrial Fibrillation and Their Clinicians: A Secondary Analysis of a Randomized Clinical Trial
Source: JAMA Netw Open. 2021 Jul 13;4(7):e2116009. doi: 10.1001/jamanetworkopen.2021.16009 (PMC8278261; doi:10.1001/jamanetworkopen.2021.16009)
Supplement: Supplement 2. — eFigure. Part of SDM Tool Highlighting Cost of Anticoagulation Options eTable. Examples of Conversation Content [file jamanetwopen-e2116009-s002.pdf]

Supplemental Online Content

Kamath CC, Giblon R, Kunnemann M, et al; Shared Decision Making for Atrial Fibrillation (SDM4AFib) Trial Investigators. Cost conversations about anticoagulation between patients with atrial fibrillation and their clinicians: a secondary analysis of a randomized clinical trial. *JAMA Netw Open.* 2021;4(7):e2116009. doi:10.1001/jamanetworkopen.2021.16009

eFigure. Part of SDM Tool Highlighting Cost of Anticoagulation Options

eTable. Examples of Conversation Content

This supplemental material has been provided by the authors to give readers additional information about their work.

© 2021 Kamath CC et al. *JAMA Network Open.*

eFigure. Part of SDM Tool highlighting cost of anticoagulation options.

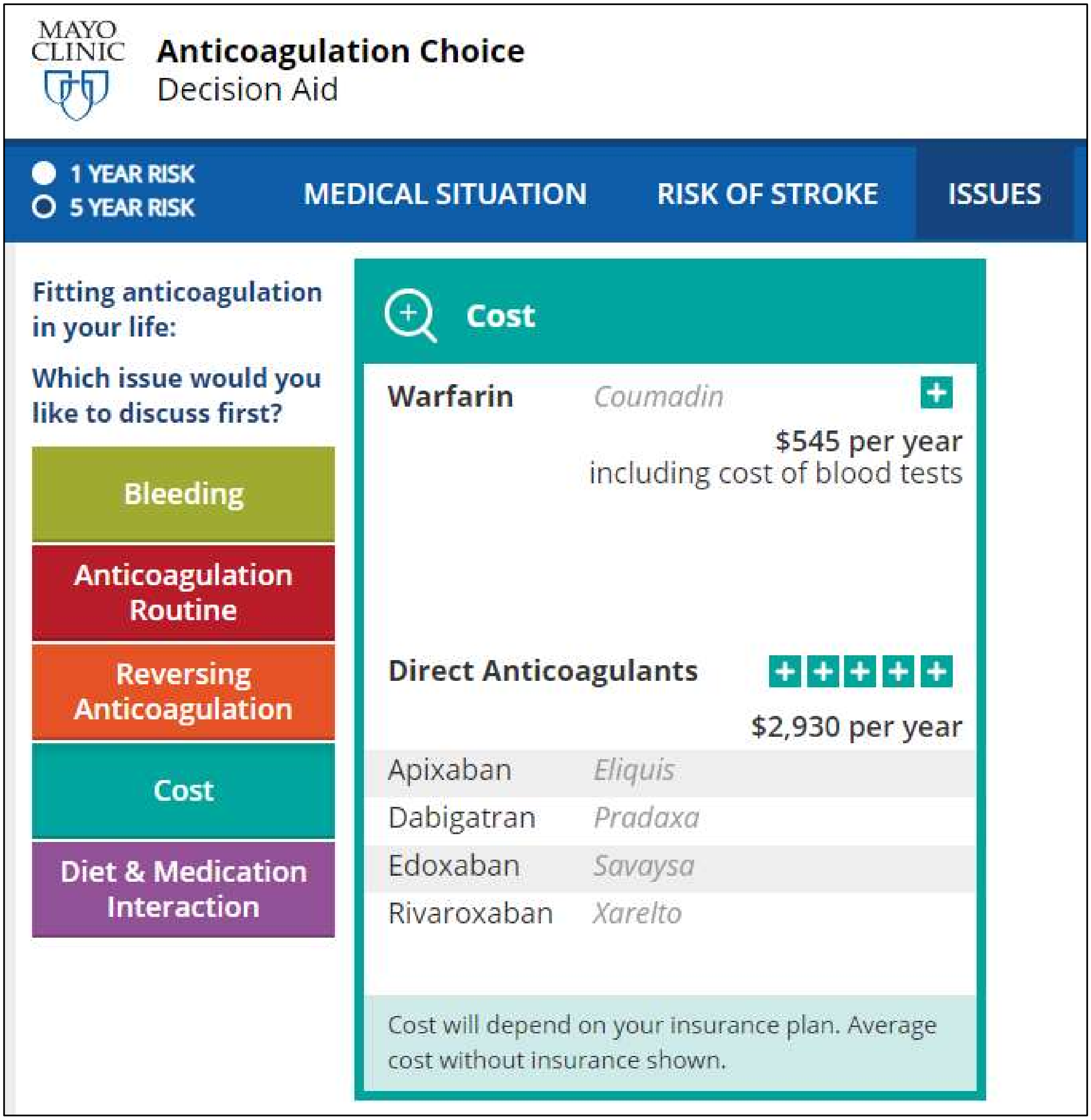

© 2021 Kamath CC et al. *JAMA Network Open.*

eTable. Examples of Conversation Content

eTable<br>**Examples of Conversation Content**
**Clinician**: Okay, so I think you're fine to stay on the Pradaxa. You could go on the company website and see if they have any discount—<br>**Patient:** Actually, we did get it.<br>**Clinician:** You did? Oh good.<br>**Patient:** My first one was free. Was it 2,000—(cross talk) Twenty-four hundred for a year. (cross talk)<br>Maybe once that coupon runs out, I'll go on something else, but then I've been home when I could be checked. Maybe after if I have an ablation maybe I won't need to be—do I really have to be on forever? Okay.<br>**Clinician:** Yeah. With a score of two, you're at moderate to high risk of developing a clot and having a stroke.[Crosstalk…]<br>When you get to a two or higher, we do recommend lifelong anticoagulation.[Crosstalk…]<br>I know. Now Eliquis, they've got a 24 month discount card where you can get it for $10 a month.[Crosstalk]<br>You can reapply for the Pradaxa one.<br>**Patient**: The pharmacist said that the Cardizem interferes with the other two. That's why we like Pradaxa.<br>[Switch to other topics temporarily]<br>It just depends on your insurance coverage.<br>**Patient**: Right. [unintelligible]. (The Health plan) owns us. (cross talk) Four more years to Medicare.<br>**Clinician**: [Laughter] yeah, my mother-in-law just went on that. [Crosstalk]<br>**Patient**: Yeah, they get to decide that. [Crosstalk]<br>This time spent in your 60s, before you reach the others, extremely expensive insurance. [Crosstalk]<br>It took us almost two years to get the ablation paid for and I had one more payment on there, and then I started—I'm like, "Rats, so close."<br>**Clinician**: I have some outstanding bills to pay to this place as well. I'll just do one refill of this at this higher dose because I don't know how long you're gonna be on it. Do you want it sent to the Sandstone Pharmacy?<br>**Patient**: Yes. [Crosstalk]<br>We'll be going over there today to get the Pradaxa. I do trust you and Dr. Hunter to decide what's best for my healthcare. I don't want to say, "I don't want that and I'm only—". I'm not that way. I pray for you guys and I pray that you have the best decisions to make for me and I'm open to that.<br>**Clinician**: Okay, thank you. We'll see though. I think it really could go either way though, so see if you have any strong feelings one way or the other 'cause that might come down to you [laughter].<br>**Patient**: Okay, but I guess I feel like the ablation is something I might consider in the future—I don't know.
**Clinician**: The other one, so it's not commonly available, but there is one for Pradaxa. The cost—you probably already noticed that—it's not cheap for the newer ones, because they're brand new. Coumadin's been around a long time, so that one's pretty easy to cover.<br>**Patient**: I think if it's $40, we can do the first prescription and see if that'll get better.[Crosstalk]<br>**Patient**: Well, we could have you write a prescription for the CBXK, at Caremark for a three month supply.<br>That provides a lower cost for us. It's a mail order. That's better.<br>**Clinician**: Okay. When you got your Xarelto, was it just a short-term prescription?<br>**Patient**: We just got one. Will it tell on my bottle? ([Crosstalk]<br>Just for 30 days, you have, so now I think if you're deciding to stay on it, then if you could call in this Caremark, to CVS Caremark, they'll—a three month supply. [Crosstalk]<br>This Xarelto says 11 refills on it, actually.<br>**Clinician**: Oh—you had it good for a year, then.<br>**Patient**: Maybe the rest of 'em Dr. Gouda's gonna call 'em in.<br>Well, but that doesn't help us with the cost. We can take that in to the prescription—[Crosstalk]<br>**Patient**: CVS Caremark's has the requirement of the 90 days. They want you to fill it for 90 days, and you get a significant reduction in the cost. Then they'll just mail it to us. 'Cause we don't have a CVS Caremark in our community.<br>**Clinician**: If you want, I can do that, or do you want to think about things? Either one.
**Clinician**: And, and, again, correct me if I'm misinterpreting this, but at least right now you'd like to investigate the warfarin plus a monitor before you ever come back to thinking about the new medications, at least at this time. [Crosstalk]

© 2021 Kamath CC et al. *JAMA Network Open.*

**Family member:** Yeah, I, I think it would be good to, to do a cost comparison between what the cost is gonna be if we go with the monitor…[Crosstalk]<br>The warfarin cost is manageable…[Crosstalk]<br>But you know the medication costs, whichever one you would choose to put her on, what that cost would be in comparison.<br>**Clinician**: We can get that information for you, um, uh, quicker…[Crosstalk]<br>than the monitor cost, I think. [Crosstalk]<br>And, again, so I don't wanna push you in any, in, in either direction, um, I want you to be comfortable with your decision here on what you feel is best for you to do. Now, I, I never say forever, I always say, I always…<br>**Patient:** I'd like… I would like to get the information.<br>**Clinician**: Yeah. I always say until we meet again, and so what I would offer, especially if you stayed on the warfarin, is that we could revisit this... if you're on the warfarin and are on the monitor, we could revisit this next year to see if it's working out, and then...<br>**Family Member**: Okay.<br>**Clinician**: And then we could go through the same process as far as whether or not that's working or you would consider the new medications. I also anticipate those reversal agents will all be approved by next year, so...[Crosstalk]<br>**Clinician**: That might take one thing out of the equation for ya.<br>**Family Member:** Okay.<br>**Clinician**: All right?
**Clinician**: The next thing to consider here is cost. Coumadin costs about $545 a year. That would include the cost of your blood tests. Whereas the direct anticoagulants are more expensive around three grand a year. All this depends on your insurance, and what they pay, and what your co-pays are, and that sort of thing.<br>**Patient**: Yeah, that's a big issue for us because number one, we're on a fixed income, and in spite of what I was, it's not a high income. We don't have drug coverage with our insurance. [Crosstalk]<br>That may change, but we don't now. [Crosstalk]<br>Warfarin becomes a more attractive—<br>**Clinician**: More attractive option based on the cost. Okay. All right.
**Clinician**: Yeah. I guess the concern would be is if you took something this expensive, that might—<br>**Patient**: That might be a concern, and I think the times that we have looked at that, that's where my big concern is, is the cost. I don't see any sense in changing if I'm getting along pretty well, I guess.<br>**Clinician**: I don't see any reason to tell you you should change. I'm making you aware of this because they're becoming more and more popular. I think there are circumstances where they are a better treatment for some patients, poorly controlled Warfarin Patients. There are some circumstances where really the Warfarin's a drug that a patient should be on, especially if they have a heart valve condition or some Patients with kidney disease who are a little more leery about the new drugs and such. All in all, you're comfortable where you're at and you wanna stay on the Warfarin for now?<br>**Patient**: Oh yeah. Like I say, the big factor for us, I think at least for me, was the cost, and this is not that inconvenient as long as it's once every five weeks, well, then I gotta get checked, that's fine. I don't have a problem with that.<br>**Clinician**: Right. Yeah, well, you're doing very, very well with your time and range and by the graph and everything, you're at a really steady state, so—<br>**Patient**: It's convenient for us. It's not that big a deal, so—
**Clinician:** -- so we have to pick, you got to tell me what's your preference. The cost wise, the newer one cost $480 per month. Warfarin is lot less expensive. [Crosstalk]<br>However, you have to think about the clinical visit, the machine, the test strip, all those kind of things and the time you're coming. And then, the newer medication, you have Humana, so, Humana, if we, if I have to do that, then what I can do is, we can send the insurance—send a letter to insurance company, ask them to pay for it. It's the same thing as they covered the machine, look at your INRs, that's really, that's pretty dangerous.<br>**Patient**: Oh, I know. Right now, yeah.<br>**Clinician**: Yeah. You have some really high ones and then very low ones. And, given the cirrhosis edema you're having, that's not good sign. So, I'm pretty confident if we apply for your insurance, ask for prior authorization, they will cover you [Crosstalk]<br>You're out of range most of the time.<br>**Patient**: yeah, I don't know, why it's jumping around so much.

**Clinician**: Yeah. If you're jumping around that much, I just don't know how much the machine's gonna help you. Just because, even if you test it, it's still going to—[Crosstalk]<br>**Patient**: I mean, a part of me is like, I just figures Coumadin's tried and true, it's been [Crosstalk] And so, I don't know. I don't know which way to go, I guess I could ask your opinion. [Crosstalk]<br>**Clinician**: But, based on my clinical experience, those newer anti-coagulant tolerate by majority patient.<br>**Patient:** Okay.<br>**Clinician**: Okay.
**Clinician**: The other one, so it's not commonly available, but there is one for Pradaxa. The cost—you probably already noticed that—it's not cheap for the newer ones, because they're brand new. Coumadin's been around a long time, so that one's pretty easy to cover.<br>**Patient**: I think if it's $40, we can do the first prescription and see if that'll get better.<br>**Clinician**: We could hope so.<br>**Patient**: Well, we could have you write a prescription for the CBXK, at Caremark for a three month supply. [Crosstalk] That provides a lower cost for us. [Crosstalk]<br>It's a mail order. That's better.<br>**Clinician:** Okay. When you got your Xarelto, was it just a short-term prescription? [Crosstalk]<br>Patient: Just for 30 days, you have, so now I think if you're deciding to stay on it, then if you could call in this Caremark, to CVS Caremark, they'll—a three month supply.<br>**Patient:** This Xarelto says 11 refills on it, actually.<br>**Clinician**: Oh—you had it good for a year, then. [Crosstalk].<br>**Patient**: Well, but that doesn't help us with the cost. We can take that in to the prescription—<br>**Clinician**: Oh, I see. Patient: CVS Caremark's has the requirement of the 90 days. They want you to fill it for 90 days, and you get a significant reduction in the cost. Then they'll just mail it to us. 'Cause we don't have a CVS Caremark in our community.<br>**Clinician**: If you want, I can do that, or do you want to think about things? Either one.

© 2021 Kamath CC et al. *JAMA Network Open.*
