# Supplementary material for: Cost Conversations About Anticoagulation Between Patients With Atrial Fibrillation and Their Clinicians: A Secondary Analysis of a Randomized Clinical Trial
Source: JAMA Netw Open. 2021 Jul 13;4(7):e2116009. doi: 10.1001/jamanetworkopen.2021.16009 (PMC8278261; doi:10.1001/jamanetworkopen.2021.16009)
Supplement: Supplement 3. — Nonauthor Collaborators [file jamanetwopen-e2116009-s003.pdf]

Supplemental Online Content: Nonauthor Collaborators

*Indicates required information. Only first name, last name, and suffix will appear in PubMed.

*Group Name(s): Shared Decision Making for Atrial Fibrillation (SDM4AFib) Trial Investigators							
*First Name and Middle Initial(s)	*Last Name	*Suffix (eg, Jr, III)	Academic Degrees	Institution	Location (city, state/province, country)	Role or Contribution, eg, chair, principal investigator	Group (if more than 1 Group listed in the byline) and/or Subgroup (eg, Steering Committee)
Alexander	Haffke			Hennepin Health	Minneapolis, Minnesota	Study Coordinator	
Amy	Stier			Mayo Clinic	Rochester, MN		
Anjali	Thota			Mayo Clinic	Rochester, MN	Study Coordinator	
Annie	LeBlanc			Mayo Clinic	Rochester, MN		
Benjamin	Simpson			Hennepin Health	Minneapolis, Minnesota	Study Coordinator	
Claudia	Zeballos-Palacios			Mayo Clinic	Rochester, MN		
Derek	Vanmeter			Mayo Clinic	Rochester, MN	Study Coordinator	
Emma	Behnken			Mayo Clinic	Rochester, MN	Study Coordinator	
Erik	Hess		MD	Division of Cardiovascular Disease, Univ	Birmingham, Alabama	Site Principal Investigator	
Henry	Ting			Mayo Clinic	Rochester, MN		
James	Hamilton	IV	MD	University of Mississippi Medical Center	Jackson, MS	Site Principal Investigator	
Joel	Anderson			Mayo Clinic	Rochester, MN	Study Coordinator	
Jonathan	Inselman			Mayo Clinic	Rochester, MN	Statistician	
Jule	Muegge			Hennepin Health	Minneapolis, Minnesota	Study Coordinator	
Kirsten	Fleming		BA	Mayo Clinic	Rochester, MN	Study manager	
Marc	Olive			Mayo Clinic	Rochester, MN	Study Coordinator	
Mark	Linzer		MD	Hennepin Health	Minneapolis, Minnesota	Site Principal Investigator	
Miamoua	Vang			Hennepin Health	Minneapolis, Minnesota	Study Coordinator	
Michael	Ferrara			Mayo Clinic	Rochester, MN		
Mike	Wambua			Hennepin Health	Minneapolis, Minnesota	Study Coordinator	
Paige	Organick			Mayo Clinic	Rochester, MN	Study Coordinator	
Renee	Cabalka			Mayo Clinic	Rochester, MN	Study Coordinator	
Sara	Poplau			Hennepin Health	Minneapolis, Minnesota	Study Coordinator	
Takeki	Suzuki		MD	University of Mississippi Medical Center	Jackson, MS	Site Principal Investigator	
Carol	Abullarade			Park Nicollet Health Services	St Lois Park, Minnesota	Study Coordinator	
Lisa	Harvey			Park Nicollet Health Services	St Lois Park, Minnesota		
Shelly	Keune			Park Nicollet Health Services	St Lois Park, Minnesota	Study Coordinator	
Timothy	Smith			Division of Cardiovascular Disease, Univ	Birmingham, Alabama	Study Coordinator	
Shannon	Stephens			Division of Cardiovascular Disease, Univ	Birmingham, Alabama	Study Coordinator	
Bryan	Barksdale			University of Mississippi Medical Center	Jackson, MS		
Theresa	Hickey			University of Mississippi Medical Center	Jackson, MS	Study Coordinator	
Roma	Peters			University of Mississippi Medical Center	Jackson, MS		
Memrie	Price			University of Mississippi Medical Center	Jackson, MS	Study Coordinator	
Connie	Watson			University of Mississippi Medical Center	Jackson, MS	Study Coordinator	
Douglas	Wolfe			University of Mississippi Medical Center	Jackson, MS		
Gordon	Guyatt					Data Safety and Monitoring Board Chair	
Brian	Haynes					Data Safety and Monitoring Board	
George	Tomlinson					Data Safety and Monitoring Board	
Paul	Daniels					Expert advisory panel	
Bernard	Gersh			Mayo Clinic	Rochester, MN	Expert advisory panel	

Supplemental Online Content: Nonauthor Collaborators

*Indicates required information. Only first name, last name, and suffix will appear in PubMed.

*First Name and Middle Initial(s)	*Last Name	*Suffix (eg, Jr, III)	Academic Degrees	Institution	Location (city, state/province, country)	Role or Contribution, eg, chair, principal investigator	Group (if more than 1 Group listed in the byline) and/or Subgroup (eg, Steering Committee)
Thomas	Jaeger			Mayo Clinic	Rochester, MN	Expert advisory panel	
Robert	McBane					Expert advisory panel	
						Expert advisory panel	
						Expert advisory panel	
